# *In vivo* porcine multi-omics integration identifies microbiome-driven histamine elevation and lasting gut perturbations following *Ascaris suum* infection and fenbendazole treatment

**DOI:** 10.1080/21505594.2026.2728766

**Published:** 2026-09-11

**Authors:** Fang Liu, Hannah Streett, Gloria Solano-Aguilar, Allen D. Smith, Donald Carbaugh, Joseph F. Urban, Robert W. Li

**Affiliations:** a Oak Ridge Institute for Science and Education, Oak Ridge, TN, USA; b United States Department of Agriculture (USDA), Animal Parasitic Diseases Laboratory, Beltsville, MD, USA; c USDA, Diet, Genomics and Immunology Laboratory, Beltsville, MD, USA; d USDA, Animal Genomics and Improvement Laboratory, Beltsville, MD, USA

**Keywords:** Ascaris, fenbendazole, histamine, Lactobacillus, metabolome, microbiota, multi-omics, swine

## Abstract

Ascaris roundworms impair human and swine health. While treatments using anthelmintic drugs are generally effective in eliminating worms, their effects on the gut microenvironment remain poorly understood. Here we applied integrated multi-omics to characterize infection- and treatment-associated alterations in the pig–Ascaris system. *In vitro* anaerobic cultures were conducted as supportive validation of selected observations. *Ascaris suum* infection altered microbial composition and dysregulated 182 serum and fecal metabolites, including histamine and p-cresol sulfate. Compared with time-matched uninfected controls, infected pigs treated with fenbendazole showed marked differences in gut microbial composition 13 days after confirmed worm clearance. Eleven microbial pathways were enriched in successfully treated pigs, including peptidoglycan biosynthesis and histidine metabolism, indicating that infection-associated alterations may persist after treatment. *In vitro* co-exposure of *Lactobacillus reuteri* to fenbendazole and *A. suum* proteins increased histamine production by approximately 79% at 48 h (*p* < 0.05), serving as supportive evidence of a microbiome contribution. Collectively, our *in vivo* findings support that host–microbiota–parasite interactions are multifaceted. Microbiota-derived metabolites were associated with regulation of host gene expression, such as *TFF2* and *IL8*. Microbiota plasticity allows the exploitation of the niche differentiated upon infection, resulting in the proliferation of certain *Lactobacillus* strains in treated animals. Nevertheless, interpretations of treatment effects are made cautiously given the absence of an uninfected drug-only group and the cross-sectional design. Understanding these complex interactions will be important for the design of next-generation functional anthelmintics.

## Introduction

Roundworms from the genus *Ascaris* are gastrointestinal (GI) nematodes important to human health and swine production. *Ascaris lumbricoides* infects up to one billion people globally, particularly in developing countries in tropical regions [1]. *A. suum*, on the other hand, is one of the most important helminth parasites during all major stages in the pig production system, from gilts, fatteners, to finishers. The prevalence is high, ranging from 15% in Japan [2] to 35% in the Scandinavian countries [3]. In organic or near-organic swine production systems, the prevalence of *A. suum* is much higher. For example, all pigs raised in a near‑organic production system examined at slaughter had white spots on the liver, characteristic of a recent *A. suum* infection, while 78% of them harbored *A. suum* worms [4]. The negative impact of *A. suum* infection on swine production includes up to 28% and 14% reductions in feed intake [5] and weight gains [6], respectively. Indirect impacts of *A. suum* infestation on food animal production include co-transmission of other pathogens, such as bacteria and viruses, and its potential infection on other livestock species, such as cattle [7]. Moreover, *A. suum* is closely related to the human roundworm *A. lumbricoides*. Considering similarities in the structure of the GI tract and physiology between their respective host species, pigs and humans, the pig – *A. suum* system represents an elegant model for human ascariasis [8].

Pig – *A. suum* interactions are complex and reciprocal. Infection by *A. suum* induces a local Th2-like host immune response. Chronic *A. suum* infection in pigs tends to result in a substantial suppression of inflammatory pathways in the intestinal mucosa, including the downregulation of genes encoding cytokines and antigen-processing and costimulatory molecules, whereas the *A. suum* body fluid has suppressive effects on human dendritic cells *in vitro* [9]. Further, *A. suum* worm expulsion is associated with eosinophils and intra-epithelial T cells in the pig jejunum [10]. Recently, the gut microbiota has been considered an integral part of the pig–*Ascaris* interaction [11–14]. *A. suum* infection in pigs significantly decreases various microbial diversity indices, including Chao1 richness, in a worm burden-independent manner [12]. In addition, the gut microbial structure of *A. lumbricoides* is rather unique and different from its human host [15], possibly playing a role in parasite fitness and nutrition. *A. suum* also releases antimicrobial factors, affecting gut bacterial growth and biofilm formation [16], further modifying host–parasite interactions.

Fenbendazole (FB), the active ingredient in the commercial oral dewormer Panacur, is a broad‑spectrum anthelmintic against GI nematodes in multiple species of veterinary relevance, such as pigs, horses, and ruminants. It belongs to the methylcarbamate benzimidazole drug family (another important member is albendazole). Its effectiveness against human roundworms, lungworms, and stomach worms is well documented. Recently, the effect of FB on the gut microbiota in a rodent model has been examined [17]. Notably, the changes in the microbiota induced by the anthelmintic drug under a parasite-free condition are sex-dependent, more profound in male than in female rodents. Further, the treatment results in a significant difference in β diversity. The abundance of the phyla *Verrucomicrobia* and *Actinobacteria* differs significantly between pre- and post- FB treatment. The abundance of *Actinobacteria* is reduced by the treatment, regardless of sex. The dual anthelmintic treatment using FB and ivermectin in Amur tigers also induced a significant change in the fecal microbial composition, including a reduction in the abundance of *Actinobacteria* [18]. Nevertheless, other studies suggest that FB may not have a detectable effect on microorganisms in the hindgut other than its targets in horses [19]. FB is known to have antiviral effects [20] and has been a popular molecule for drug repurposing. FB can be extensively metabolized by the cytochrome P450 isoforms in the liver [21]. Nevertheless, the gut microbes responsible for microbial biotransformation of FB are unknown. To address these knowledge gaps, we investigated interactions between the porcine gut microbiota, *A. suum*, and fenbendazole using an integrated multi-omics approach in a controlled challenge infection model. Our particular interest lies in the potential physiological and ecological consequences of successful anthelmintic treatments in pigs.

## Results

### Infection-induced alterations in the porcine gut microbiota were long-lasting

**Figure 1.**
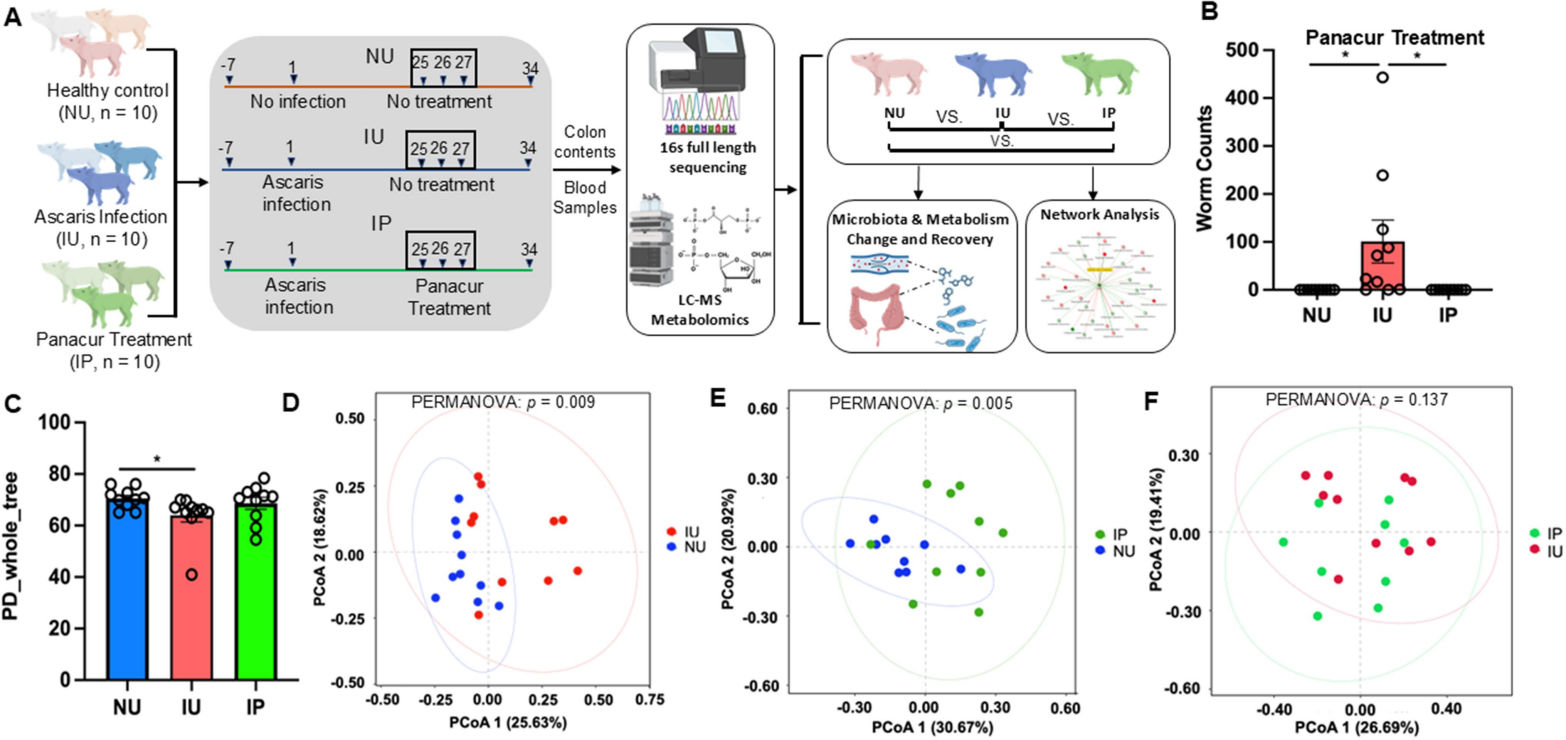
The roundworm *A. suum* infection and subsequent anthelmintic treatment by fenbendazole (Panacur) had a substantial impact on gut microbial communities in pigs. A. a schematic diagram showing the study timeline. The number represents days post inoculation (DPI). The black box showing the dates of anthelmintic drug treatment. B. the drug treatment at the recommended oral dose, 2.5 fenbendazole mg/kg bodyweight, for three consecutive days was highly effective in eliminating adult *A. suum* worms. C. *A. suum* infection significantly reduced Phylogenetic diversity (PD) whole tree. The beta diversity was also significantly different between the infected and uninfected pigs (D) or between NU and IP (E) or between IU and IP (F), although this comparison was not statistically significant (PERMANOVA *p* = 0.137). NU: Normal uninfected and untreated controls. IU: Infected and untreated pigs. IP: Infected and Panacur‑treated pigs. *p < 0.05. **p < 0.01. *N* = 10 per group.

The mean number of worms recovered at necropsy in infected (34 days post-infection or dpi) but untreated pigs (IU) was 100.9 (±44.9; SD; Figure 1(A,B)). The female/male ratio among the recovered adults was 1.95 while the mean number of the larvae was 12.7 (*N* = 10). The skewed distribution of the worms among individual animals was evident. Approximately 90% of the worms were recovered from only 40% of pigs while no worms were detected in two of these infected pigs. This substantial inter-individual variation aligns with well-documented overdispersion in helminth systems; accordingly, we interpreted microbiome and metabolome differences conservatively. At 34 dpi, the worm can grow up to 14 cm in length. Visual inspections detected white specks on the liver (milk spots) in infected pigs with healing morphology. The drug treatment with three consecutive doses of fenbendazole (Panacur) in infected animals (IP) was effective; and no worms were recovered from the gut contents of these treated animals (Figure 1(B)). As expected, no worms were seen in normal (uninfected and untreated) pigs (NU).

*A. suum* infections at 34 dpi resulted in a significant alteration in the gut microbial community in the porcine proximal colon, in agreement with the previous findings [12]. Compared to uninfected and time-matched controls (NU), the infection at 34 dpi repressed alpha diversity as measured by phylogenetic diversity (PD) whole tree (Figure 1(C)) and the Simpson index (Fig. S1A). The drug treatment partially restored the Simpson index. Infection also altered the β diversity: PERMANOVA analysis based on the Bray-Curtis dissimilarity matrix revealed a clear separation in the gut microbial communities between NU and IU groups (Figure 1(D)). Treatment-associated effects cannot be fully separated from infection resolution in the absence of an uninfected drug-only control. FB treatment did not restore the structure of the gut microbial communities in infected animals to the NU baseline (Figure 1(E)); and the community structures between drug-treated and untreated in infected animals remained indistinguishable (Figure 1(F)). Nevertheless, these observations are interpreted as associations rather than causal effects of FB.

**Figure 2.**
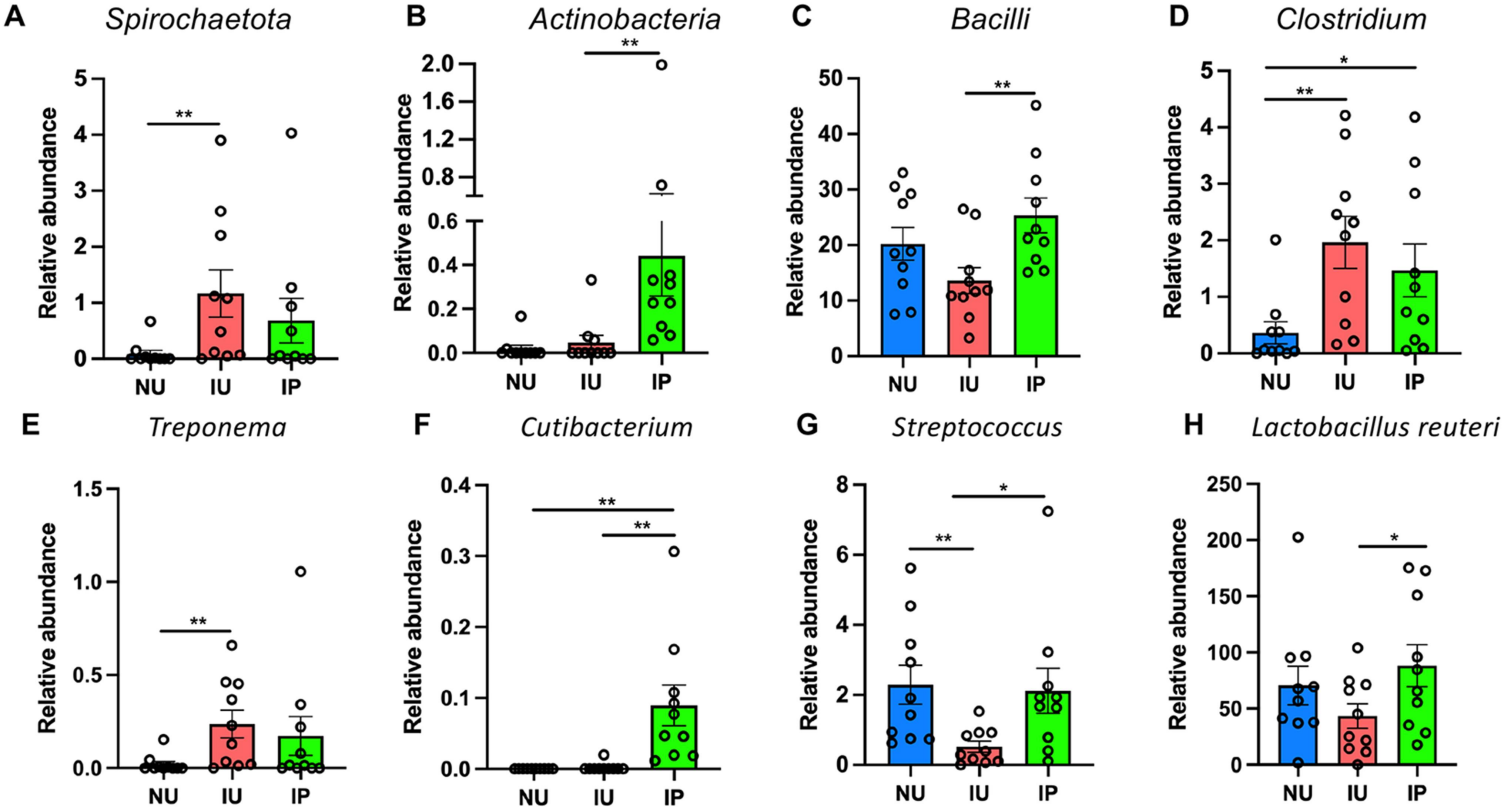
The changes in the gut microbial composition during *A. suum* infection and subsequent anthelmintic treatment. A. The phylum *Spirochaetota*. B. The class *Actinobacteria*. C. The class *Bacilli*. D. *Clostridium*. E. *Treponema*. F. *Cutibacterium*. G. *Streptococcus*. H. *Lactobacillus reuteri*. NU: Normal uninfected and untreated controls. IU: Infected and untreated pigs. IP: Infected and Panacur‑treated pigs. *p < 0.05. **p < 0.01. *N* = 10 per group.

**Figure 3.**
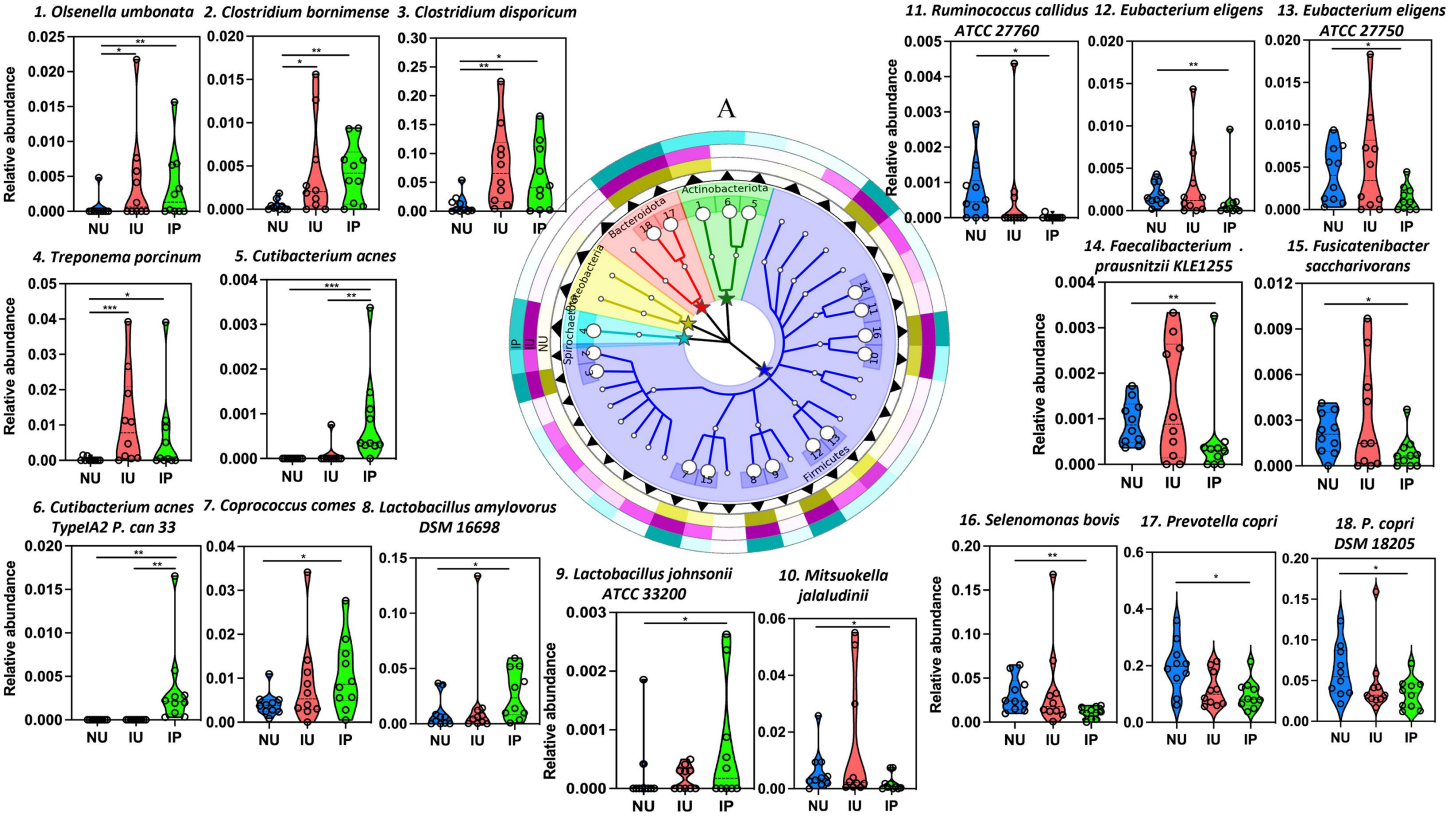
Eighteen bacterial species and strains impacted by the roundworm infection and fenbendazole treatment. A. The circular representations of taxonomic and phylogenetic trees affected by the infection and ensuing drug treatment. The numbers in the tip of the tree represent one of the 18 species significantly impacted by the infection and anthelmintic treatment. The Y-axis in the bar charts denotes relevant abundance. NU: Normal uninfected and untreated controls. IU: Infected and untreated pigs. IP: infected and the fenbendazole (Panacur) treated pigs. *p < 0.05. **p < 0.01. ***p < 0.001. *N* = 10 per group.

Infection induced a significant alteration in one of the phyla, *Spirochaetales* (*p* = 0.0021; FDR = 0.0146; Figure 2(A)). Multiple families, including *Spirochaetaceae* and *Caloramtaoraceae*, were significantly affected by infection (Fig. S1B-1F). Synthetic full-length based sequencing of the 16S rRNA gene detected 363 bacterial species or strains in the porcine gut microbiota. *Prevotella copri* (including *P. copri* strain DSM18205) and *Streptococcus alactolyticus* were among the most abundant in the gut of NU pigs. Infection also altered the abundance of 18 species and strains, representing approximately 18.4% of all species detected in individual pigs. For instance, infection reduced *S. alactolyticus* abundance 2.9‑fold, from 8.62% in uninfected controls to 2.62% in infected animals and increased *Clostridium bornimense* ~10‑fold. Although FB cleared all worms, the gut microbiome did not revert to baseline (Figure 2(B–H)). Thirteen days after the last dose of the drug treatment, at least two classes, *Actinobacteria* and *Bacilli*, differed significantly between IP and IU groups (FDR <0.05, Figure 2(B,C)). Five genera, including *Clostridium* (Figure 2(D)), *Treponema* (Figure 2(E)), *Cutibacterium* (*p* = 4.33 × 10^−5^, FDR = 0.001; Figure 2(F)), and *Streptococcus* (Figure 2(G)), differed. The abundance of *Lactobacillus reuteri* was significantly higher after the drug treatment (Figure 2(H)). Eighteen species/strains differed significantly between IP and IU pigs (Figure 3). For example, while the infection did not appear to affect the abundance of *L. reuteri* strain I5007 and *L. johnsonii* strain ATCC33200, the drug treatment significantly increased their abundance in infected pigs. The species or strains significantly impacted by the drug treatment were not randomly distributed but mainly clustered in the classes of *Actinobacteria* and *Bacilli* (Figure 3). Given design limitations (no FB-only group), we avoid attributing these differences strictly to drug effects.

### Anthelmintic drug clearance modified porcine serum metabolome

**Figure 4.**
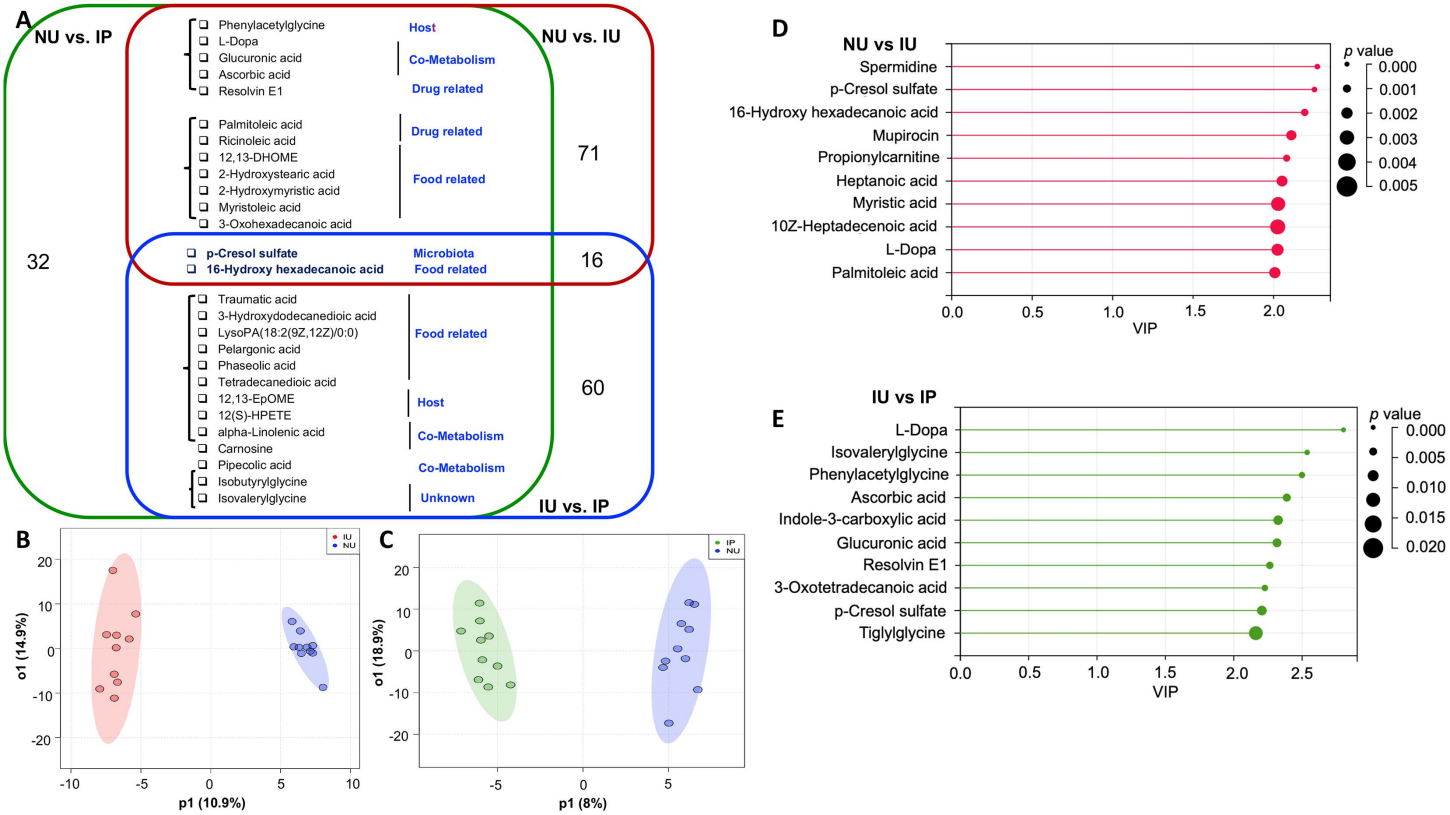
The serum untargeted metabolome analysis in *A. suum* infected pigs treated with fenbendazole. A. The summary of the serum metabolites significantly dysregulated during infection and ensuing fenbendazole treatment. The colored boxes represent comparisons between groups. The number represents the number of dysregulated metabolites at the cutoff value of FDR <0.05. The categories in the blue font represent the predicted origin of the metabolites detected. B. sparse partial least squares (sPLS) analysis showing that the groups between the uninfected and infected were clearly separated or C. between IP and NU. D. The top 10 metabolites distinguishing the infection status (NU vs IU) based on the variable importance (VIP) scores. E. The 10 most important serum metabolites distinguishing the fenbendazole treatment in the infected pigs based on VIP scores. NU: Normal uninfected and untreated controls. IU: Infected and untreated pigs. IP: Infected and the fenbendazole (Panacur)-treated pigs. *N* = 10 per group.

The infection and subsequent drug treatment (IP) induced distinct serum metabolic profiles (Figure 4(A)). PCA showed a clear separation between IU and NU (Figure 4(B)) and between IP and NU (Figure 4(C)). At least 106 metabolites differed significantly due to infection and/or drug treatment (*p* < 0.05 and fold change FC >1.50). For example, compared to uninfected controls, the serum level of pimelic acid, which was detected in both positive and negative electrospray ionization modes (EMI), isobutyric acid, and p-cresol sulfate, all of microbiota origin, were significantly higher in IU pigs. Mupirocin, resolvin E1, and spearmidine were also ~2-fold higher in infected pigs. The top 10 metabolites distinguishing the infection status (NU vs IU) based on the variable importance (VIP) scores included spermidine, p-cresol sulfate, 16-hydroxyhexadecanoic acid, and mupirocin (Figure 4(D)). Random Forests (RF) analysis confirmed mupirocin and p-cresol sulfate as top classifiers (Fig. S2A). By contrast, dozens of fatty acids (e.g. docosapentaenoic acid, linoelaidic acid, oleic acid) were repressed by infection (Table S1). Drug treatment in infected pigs (IP) was associated with the increased serum level of at least seven metabolites (*p* < 0.05; FC > 1.50), including γ-glutamylvaline, which were among the most important metabolites distinguishing the drug treatment (Figure 4(E); Fig. S2B). On the other hand, isobutyric acid, p-cresol sulfate, and salicylic acid were significantly lower in IP compared to IU. Twenty‑seven metabolic pathways were significantly enriched among FB-associated metabolic changes, including linoleic acid metabolism and arachidonic acid metabolism (host origin) and phenylalanine metabolism (microbiota origin). Nevertheless, interpretations remain associative due to the absence of an uninfected drug-only control.

### Fecal metabolites dysregulated by infection and drug treatment

**Figure 5.**
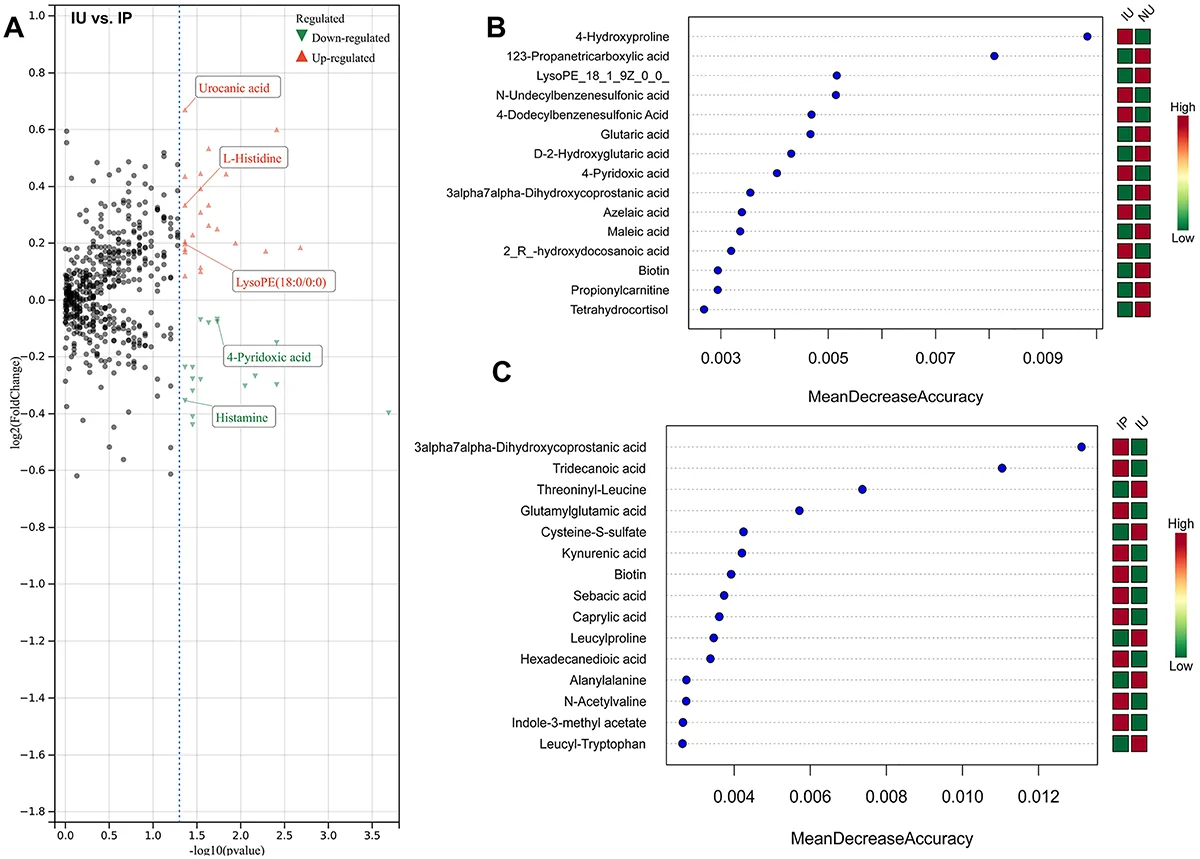
The volcano plot that shows the serum metabolites significantly dysregulated by infection and fenbendazole treatment. A. The metabolites differ significantly between the infection status. B. The serum metabolites remained dysregulated 13 days after the last dose of the fenbendazole drug treatment. X-axis: *p* value (log_10_). Y-axis: Fold change (log_10_). NU: Normal uninfected and untreated controls. IU: Infected and untreated pigs. IP: Infected and the fenbendazole (Panacur) treated pigs. *N* = 10 per group.

*A. suum* infection had a notable impact on the fecal metabolome, which was different from the serum metabolome, affecting a total of 76 metabolites at a combined cutoff of *p* value < 0.05 and FC ≥1.50. Succinic acid, 4-pyridoxic acid, stigmasterol, and azelaic acid were significantly higher in IU pigs. p-Cresol sulfate was also higher in the feces of infected animals (*p* = 0.0355; FC = 1.8). Conversely, LysoPE (18:1), naphthalene, and adenine were lower. FB treatment (IP) did not restore the dysregulated metabolites to the NU baseline. At least 31 fecal metabolites remained significantly altered in IP versus IU (Figure 5(A)). Notably, fecal histamine was significantly higher in IP pigs, unlike the serum results. Oleic acid and D-xylose were among the metabolites significantly dysregulated (*p* < 0.05; FC >2.0). L-histidine, the substrate of histamine biosynthesis, histidinyl-leucine, and urocanic acid were significantly reduced by treatment. MetOrigin assigned host, microbial, or mixed origins for dysregulated metabolites [22]. RF identified major discriminative metabolites (Figure 5(B,C)). For example, 4-hydroxypoline was the most important metabolite distinguishing the uninfected and infected pigs while 3α,7α-dihydroxycoprostanic acid, a bile acid precursor, and tridecanoic acid were the two most discriminative metabolites on the drug treatment (IU versus IP). Ten pathways were significantly enriched among the infection-dysregulated metabolites; 11 among drug-associated metabolites. (*p* < 0.05; Table S2). Among the pathways impacted, D-alanine metabolism and peptidoglycan biosynthesis were microbiota origin while β-alanine metabolism and primary bile acid biosynthesis, and histidine metabolism and biotin metabolism were of host and mixed origin, respectively.

Gas chromatography analysis showed that infection did not affect total SCFAs (Fig. S3), whereas FB restored fecal butyrate level to the NU baseline (*p* < 0.05). Correlations between metabolites and microbial species varied across groups (Fig. S4). In NU pigs, *Fusicatenibacter saccharivorans* had a strong positive correlation with fecal L-histidine level (*r* = 0.87; *p* = 0.0027). On the other hand, *L. johnsonii* ATCC 33,200 was positively correlated with fecal histamine levels in infected and FB‑treated pigs (*r* = 0.82; *p* = 0.0036).

### Multi-omics data integration using DIABLO

**Figure 6.**
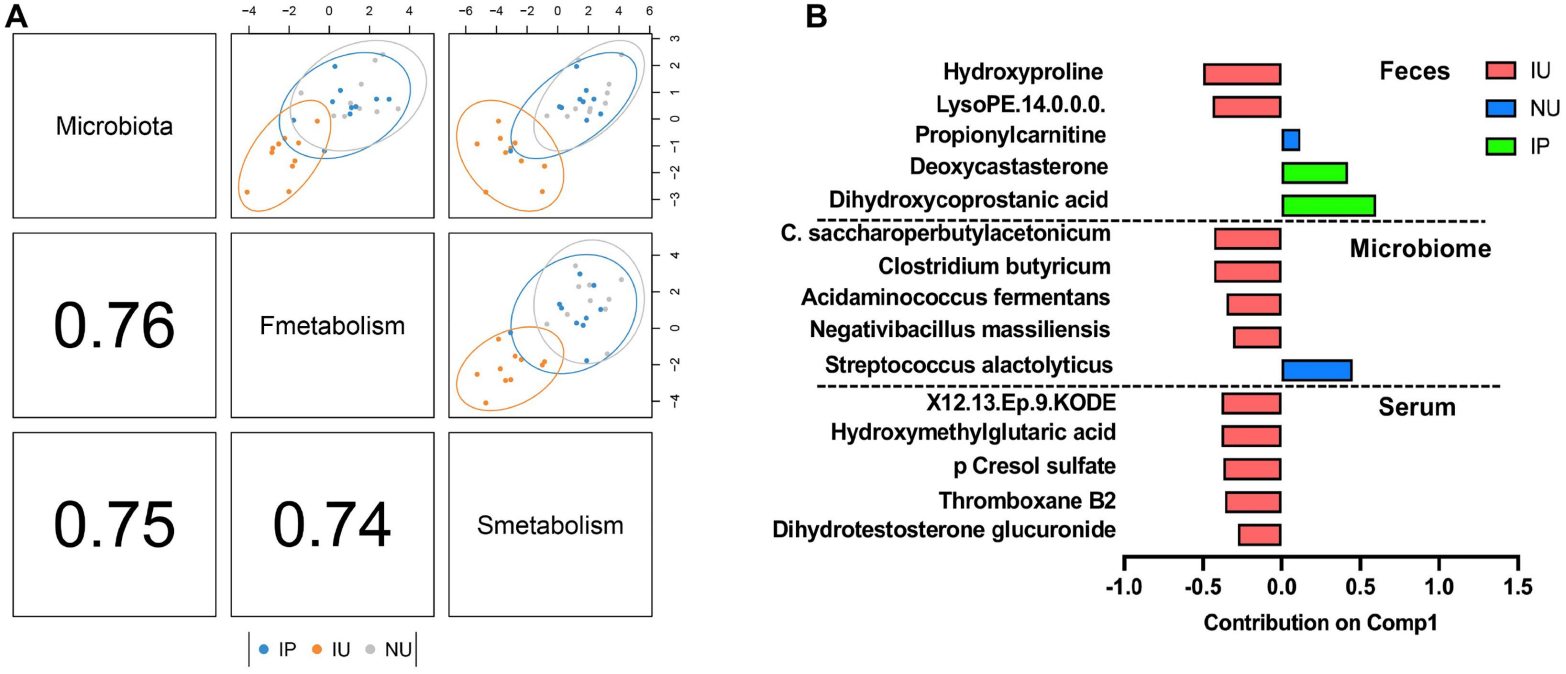
The integrated multi-omics analysis using the DIABLO algorithm identified the most important features in each data block contributing to the first component. A. a diagnostic plot based on sparse partial least squares discriminant analysis (sPLS-DA) showing that the 1st components from each data block, fecal metabolome, serum metabolome, and fecal microbiome, were moderately correlated. The number in the bottom left indicates the correlation coefficients between the first component from each data block. The ellipses represent 95% confidence. The centroids between uninfected and untreated (NU) and the infected and fenbendazole‑treated groups were highly overlapped, which were distinct from that of the infected and untreated sample group in their confidence ellipses (IU). B. The top features or variables based on the absolute value of their coefficients that strongly contribute to the first component. The variables were selected using multiblock sPLS-DA. Fmetabolism: fecal metabolome; smetabolism: serum metabolome. Microbiota: fecal microbiome. NU: Normal uninfected and untreated controls. IU: Infected and untreated pigs. IP: Infected and fenbendazole (Panacur) treated pigs. *N* = 10 per group.

**Figure 7.**
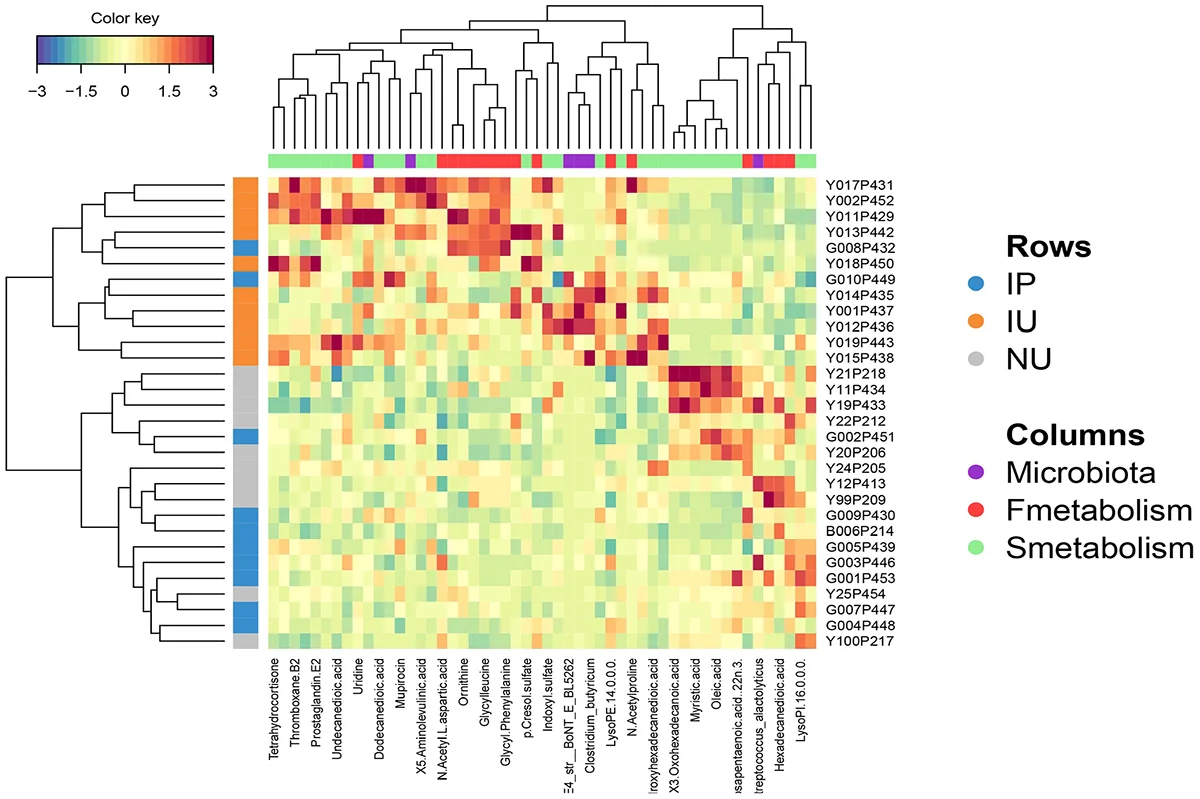
The clustered image map depicted the molecular signatures with discriminating power for each sample type selected using multiblock sparse partial least squares discriminant analysis (sPLS-DA). The samples are in rows while selected features or variables are in columns. The color key represents relative values. The top of the plot indicates clustering of samples; the colored bar below denotes data block types. Fmetabolism: fecal metabolome; Smetabolism: serum metabolome. Microbiota: fecal microbiome. NU: Normal uninfected and untreated controls. IU: Infected and untreated pigs. IP: Infected and the fenbendazole (Panacur) treated pigs. *N* = 10 per group.

DIABLO integration of microbiota, fecal metabolome, and serum metabolome identified discriminative molecular signatures [23]. A sample scatterplot depicted Spearman’s correlations between the first component of the three data blocks (Figure 6(A)). The first component of each data block was modestly correlated with each other (*r* ≥0.74). The IU group was clearly separated, while IP showed partial overlap with NU (Fig. S5). For example, the correlation between the microbiome data block (Microbiota) and the fecal metabolomics data block (Fmetabolism) was 0.76, slightly higher than the correlation between the two metabolome data blocks (Fmetabolism and Smetabolism). The discriminative power of the component to separate the experimental groups was strong. For example, while there existed a substantial overlap between NU and IP groups, the IU group had a distinct first component centroid value, suggesting that the drug treatment in infected pigs resulted in the partial restoration of the microbiome composition to the baseline observed in the normal uninfected pigs (Fig. S5). This partial restoration is interpreted cautiously and does not imply full normalization due to the cross-sectional design. The circos plot (Fig. S6) presented an overview of correlations between variables of different data blocks, highlighting species such as *Cutibacterium acnes* and serum metabolites such as LysoPE (15:0/0:0). The latter was positively correlated with the fecal metabolite LysoPA (18:1(9Z)/0:0) (*r* = 0.82). The blocks of homogeneous colors presented subsets of selected variables from each dataset that were correlated. For example, a group of metabolite features, including tetrahydrocortisone, thromboxane B2, prostaglandin E2, undecanedioic acid, uridine, dodecanedioic acid, mupirocin, 5-aminolevulinic acid, N-acetyl-L-aspartic acid, ornithine, glycylleucine, and glycyl-Phenylalanine, were discriminative against the IU group, indicating potential causal relationships. On the other hand, at least three serum metabolites, 3-oxohexadecanoic acid, myristic acid, and oleic acid, were important in the normal uninfected group. Of note, *S. alactolyticus* was among the important contributors to the first component in the microbiome data block in the uninfected control pigs (Figure 6(B)). Further, a clustered image map (heatmap) with a group of variables selected by the sPLS-DA model that possessed discriminative power between the three experimental groups was presented in Figure 7. For example, a small group of metabolites (top left) was strongly discriminative against the infected but untreated pigs.

### Effect of fenbendazole treatments on host ileal gene expression

**Figure 8.**
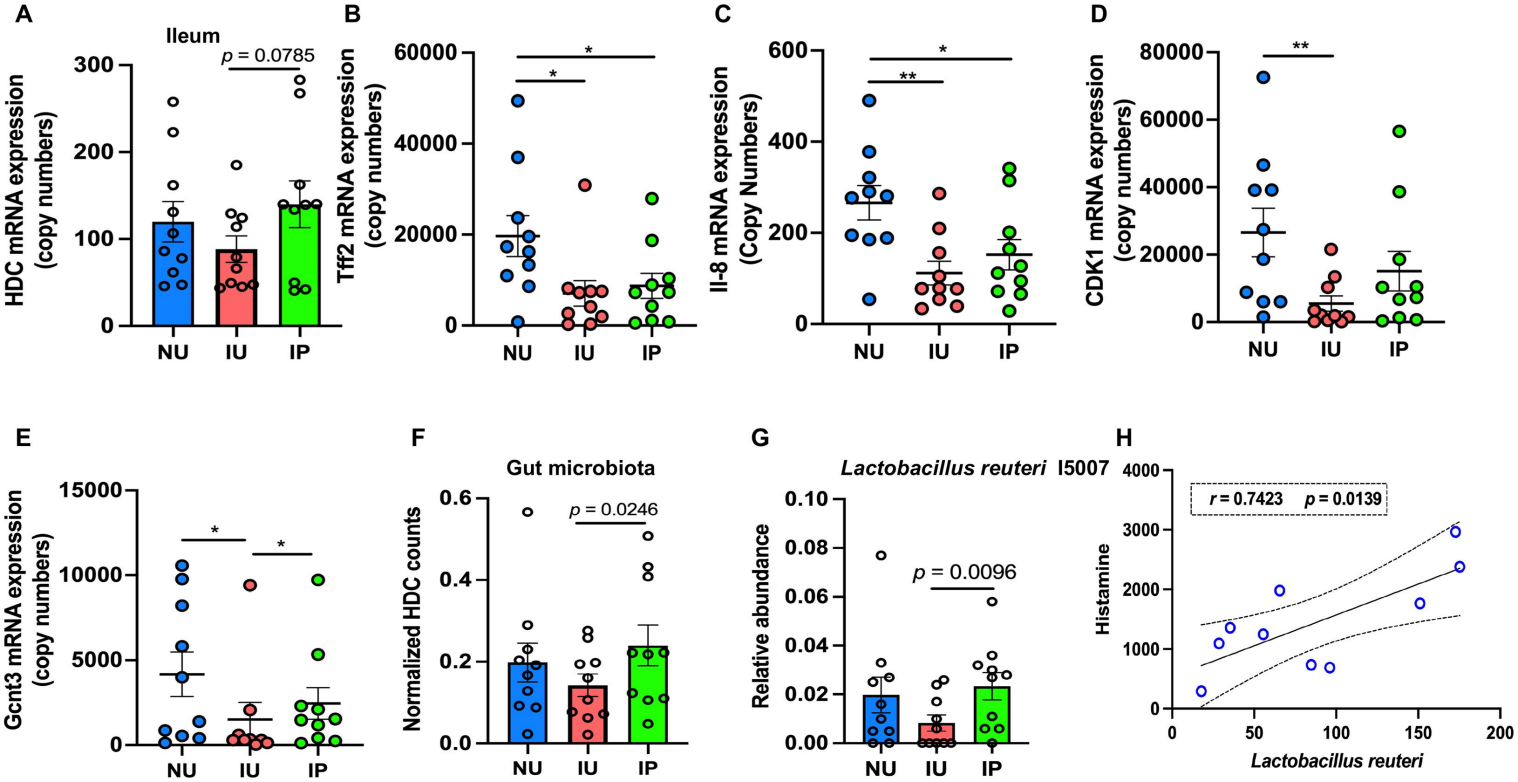
The expression of selected genes in ileum tissue and relative abundance of the histidine decarboxylase (*hdc*) gene that encodes a member of the group II decarboxylase family that converts L-histidine to histamine in the gut microbiota. A. The host *Hdc* gene expression in the ileum tissue. The expression at the mRNA level in the ileum tissue: B. Trefoil factor 2 (*Tff2*); C. Interleukin 8 (*Il8*). D. Cyclin-dependent kinase 1 (*Cdk1*); and E. Glucosaminyl (N-acetyl) transferase 3, mucin type (*Gcnt3*). F. Collective counts of the *hdc* gene of microbial origin in the gut microbiota. G. Relative abundance of *Lactobacillus reuteri* I5007. H. Correlation between the relative abundance of *Lactobacillus reuteri* and fecal histamine levels. NU: Normal uninfected and untreated controls. IU: Infected and untreated pigs. IP: Infected and Panacur (fenbendazole) treated pigs. *p < 0.05. **p < 0.01. ***p < 0.001. *N* = 10 per group.

To understand the potential effect of the anthelmintic drug treatment on the host tissue, we conducted a real-time RT‑PCR analysis of multiple genes in the total RNA extracted from the ileum tissue collected at necropsy (34 dpi). These genes included those involved in innate immunity, such as glucosaminyl (N-acetyl) transferase 3, mucin type (*GCNT3*), interleukin 8 (*IL8*), and toll-like receptor 4 (*TLR4*), tissue remodeling and cell cycle regulation, such as trefoil factor 2 (*TFF2*) and cyclin-dependent kinase 1 (*CDK1*), histamine biosynthesis and function, such as histidine decarboxylase gene (HDC) and histamine receptor H1 (*HRH1*) and a mast cell marker, tryptase beta 2 (TPSB2). *A. suum* infection and ensuing drug treatment did not appear to have any effect on the expression of *HDC* in the host tissue at the mRNA level (Figure 8(A)). However, both events had a significant effect on the expression of *TFF2*, an important factor in mucosal repair and protection in digestive and respiratory systems as well as tissue remodeling (Figure 8(B)). The infection significantly downregulated *TFF2* mRNA level (z-score = 2.6080; two-tailed Wilcoxon *p* = 0.0091). The drug treatment numerically increased its expression but failed to reach a statistically significant level. The infection also downregulated *IL8* expression (*p* = 0.0017; Figure 8(C)). The drug treatment improved the *IL8* level repressed by infection but only numerically. Infection also significantly downregulated the expression of *CDK1* by 4.8 fold (Figure 8(D)). However, compared to infected but untreated animals (IU), its mRNA level was 2.7 fold higher in the infected and drug‑treated pigs marginally (*p* < 0.1). While its mRNA expression was relatively low, the infection seemingly downregulated the abundance of HRH1 (*p* = 0.0036), compared to uninfected controls. The expression of a key gene involved in mucin biosynthesis, *GCNT3*, followed a similar trend, repressed by infection and marginally increased due to the drug treatment in infected pigs (Figure 8(E)). Of note, there existed substantial variations in the mRNA abundance of these genes among individual pigs.

In addition to the *HDC* gene in the host tissue, we also looked at the histidine decarboxylase gene (*hdc*) of microbial origin in the fecal microbiota. Collectively, the *hdc* copy number was significantly higher in the gut microbiome of FB‑treated pigs (Figure 8(F)). The abundance of both *L. reuteri* and *L. reuteri* strain I5007 was significantly increased by FB treatment of infected pigs, compared to infected but untreated group (IU) (Figure 8(G)). The former had a significant positive correlation with fecal histamine levels (Figure 8(H)).

### Histamine production by *Lactobacillus* reuteri was enhanced by fenbendazole in the presence of *A. suum* proteins *in vitro*

**Figure 9.**
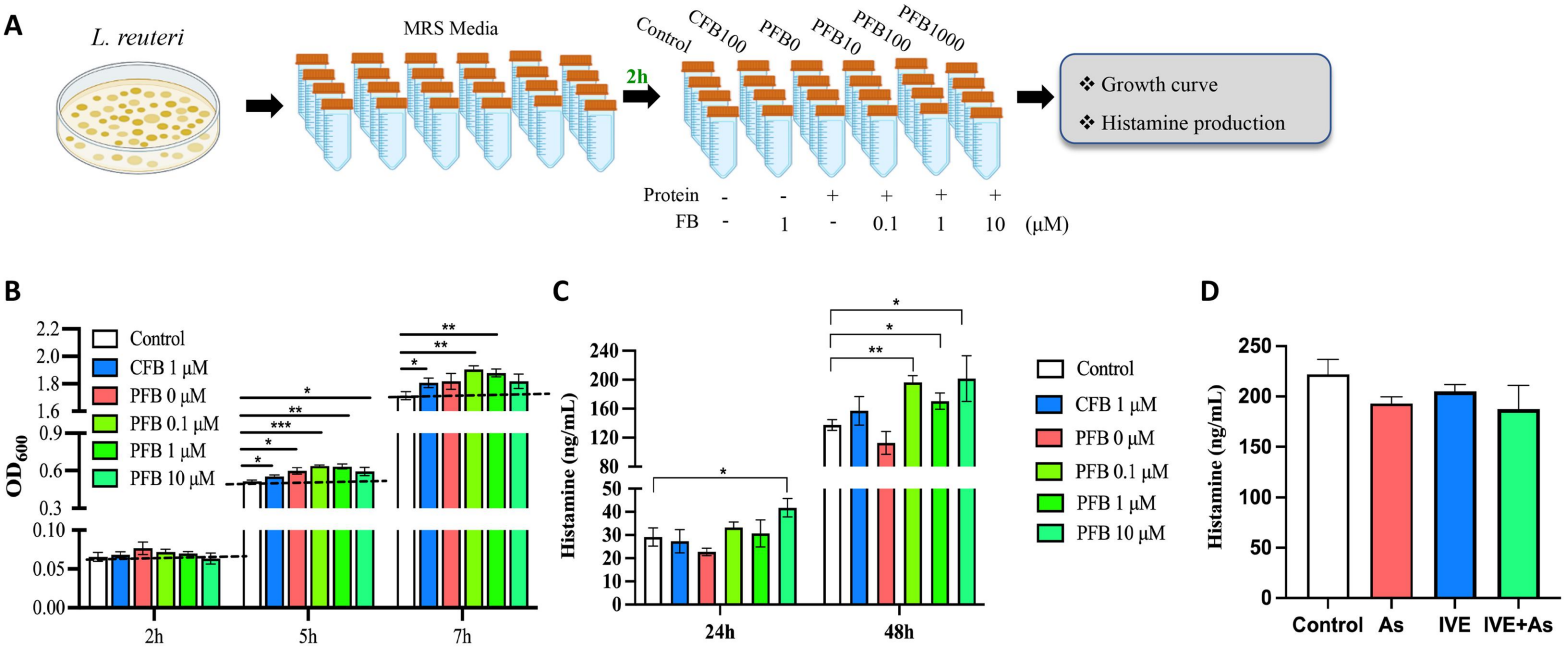
The histamine production by *L. reuteri* and porcine fecal gut microbes under aerobic and anaerobic conditions, respectively. A. Schematic culture setup. B. *L. reuteri* growth under aerobic conditions as judged by optical density (OD) at 600 nm wavelength (OD_600)._ C. Amount of histamine in culture media detected using a histamine ELISA kit. D. Histamine production in culture media of pig fecal culture under anaerobic conditions. As or Protein: *A. suum* crude proteins. FB: fenbendazole. IVE: ivermectin. CFB: FB without *Ascaris* proteins. PFB: FB with *Ascaris* proteins. Each datapoint represents four replicates. *p < 0.05. **p < 0.01. ***p < 0.001.

*L. reuteri* strain F225, a known hyper-histamine producer, exposed to both FB and *A. suum* proteins exhibited significantly increased histamine production under aerobic conditions (Figure 9(A)). Moreover, *A. suum* crude proteins and FB, alone or in combination, appeared to stimulate *L. reuteri* growth starting at 5 h culture, judged by a significant increase in OD600 values (Figure 9(B)). Due to strong auto-aggregation properties of this strain, a longer culture did not induce any additional stimulatory effects. *A. suum* crude proteins alone had no effects on histamine production by *L. reuteri*, either at 24 h or 48 h culture. Neither did FB alone. However, histamine production by *L. reuteri* was strongly enhanced by FB in the presence of *A. suum* crude proteins (Figure 9(C)). For example, compared to the culture in the presence of *A. suum* proteins but without any FB, 10 µM FB treatment increased histamine concentration in the culture media by 83.7% and 78.5% at 24 h and 48 h, respectively (*p* < 0.05). It appears that lower FB dose levels, even at 0.1 µM, also strongly stimulated histamine production (*p* < 0.01), but only in the presence of *A. suum* proteins. Together, these data demonstrate that the interactions between *A. suum* proteins and FB can have a profound impact on the histamine‑producing bacteria and histamine biosynthesis in the gut. Nevertheless, this impact is likely drug specific. Under anaerobic conditions, the histamine concentration in the supernatant of the pig fecal inoculum did not change by another widely used anthelmintic drug, ivermectin, at the equivalent dose level, with or without *A. suum* proteins (Figure 9(D)).

## Discussion

Roundworms are important to human health and swine production. The cross‑infectivity between *A. lumbricoides* and *A. suum* in their respective hosts, humans and pigs, is well documented [24]. In healthy human subjects, experimental infection with *A. suum* eggs demonstrates that *A. suum* is able to complete its life cycle in humans and results in clinical symptoms similar to those caused by *A. lumbricoides* and elicits similar immune responses. Considering the structural and physiological resemblance in the GI tract between the two host species, *A. suum* infections in pigs represent a suitable model to study ascariasis in humans and have been used to develop novel antiparasitic strategies [25]. Our study leverages this porcine model to investigate infection- and treatment-associated alterations at the systems level *in vivo*.

*A. suum* infections in pigs are known to induce a significant alteration in the structure and function of the gut microbiota [12]. The infection significantly decreased gut microbial richness in a worm-burden independent manner. The infection is also associated with a significant shift in the metabolic potential of the gut microbiome and altering the abundance of at least 30 metabolic pathways, including carbohydrate and amino acid metabolism. Several subsequent studies also validated the effect of the infection on the gut microbiota [14,26]. A single-dose inoculation of *A. suum* eggs in pigs causes a remarkable but transient decrease in microbial diversity, followed by a fluctuation in SCFA profiles and a decrease in SCFA production at 35 dpi [14]. Other studies show that both *A. suum* infection and proanthocyanidins supplementation can alter gut microbial compositions, but in a distinctly different manner [26]. In our study, *A. suum* infection at 34 dpi resulted in a significant increase in the abundance of the phylum Spirochaetota, as well as 18 species (representing ~18.3% of all detected species) compared with uninfected control pigs. The potential ecological roles of Spirochaetota in shaping host–microbiota–parasite interactions warrant further investigation but remain to be clarified.

Many other members of the family *Ascarididae* are also relevant to health and wellness of humans and animals. Among the ascarid species that infect horses, *Parascaris univalens* is common, whereas *P. equorum* is the most prevalent. Clinical symptoms of *Parascaris* infections in juvenile horses include coughing, lethargy, poor appetite, diarrhea, nasal discharge, liver and lung lesions, and impaction colic. The microbial composition of different organs of *Parascaris* worms collected from foals raised on pasture, along with gut contents sampled from the host, was characterized [27]. Eleven genera, such as *Lactobacillus*, *Sarcina*, *Streptococcus*, and *Veillonella*. appear to be common in the gut of *Parascaris* worms, regardless of their sexes. Moreover, there exists a significant difference in the Shannon index between the microbiota of parasite female intestine and that of the host jejunum. At least two ascarids, *Toxocara canis* and *Toxascaris leonine*, infect dogs and can be zoonotic. Recently, the effect of *T. canis* infection and anthelmintics has been examined in dogs [28,29]. *T. canis* infection decreased α diversity, in consistent with our findings. Furthermore, anthelmintic treatment increases α diversity and the abundance of *Segatella* [29]. Similar effects of infection on the gut microbiome were also observed in humans infected with *A. lumbricoides* [30]. The gut microbial communities between infected and uninfected human subjects were distinctly different based on the β diversity. The former have significantly reduced α diversity and higher abundance of *Proteobacteria* than the latter [30]. The infection by *A. lumbricoides* in children decreases species richness and induces a perturbation in interaction networks, resulting in a loss of interactions of *Coprococcus* with other families of bacteria and contributing to the emergence of depression [31]. Further, the gut of *A. lumbricoides* harbors a gut microbiota profile distinct from its human host [15]. Metabolomic profiles of *A. lumbricoides* worms isolated from patients with heavy and light ascariasis cases differ significantly. Multiple metabolites, such as ethanesulfonic acid, apicidin, and aripiprazole lauroxil, are significantly increased in worms isolated from patients with heavy ascariasis, resulting in an enrichment of several pathways, including those related to α-linolenic acid metabolism and steroid biosynthesis [30]. Nevertheless, one of the common shortcomings of these studies on ascarids is that only 16S rRNA gene sequencing based on selected few hypervariable regions, such as V3-V4 regions, was used, which can differentiate bacteria only at the genus level. In the present study, we utilized the synthetic long read technology on the Illumina platform so that the full-length 16S rRNA gene sequences can be generated, which allows the identification of gut microbes at species and strain levels based on sequence homology search. Our present study is also unique in several other aspects. First, we conducted an experimental infection in a controlled environment with a synchronized stage of infection, minimizing the variations associated with temporal and worm developmental factors. We also validated our key findings independently using an *in vitro* pure culture system. In addition, we utilized an integrated multi-omics pipeline in data analysis. The advanced algorithms in the pipeline have proven powerful in detecting previously unrecognized biological interactions, allowing us to dissect molecular mechanisms implicated in the manifestation of *A. suum* pathology. Despite these strengths, our experimental design lacked an uninfected fenbendazole-only group. Moreover, the lack of noninvasive biopsy techniques prevented us from serial sampling of proximal colon contents. Otherwise, a longitudinal study with repeated sampling of the same variables over multiple time points would be powerful in understanding dynamics of gut microbiota perturbation induced by *A. suum* infection and its physiological and pathological consequences, helping us pinpoint causal relations in host-microbiota-parasite interactions [32]. These constraints limit causal inference; we therefore interpret treatment-associated differences with caution and frame mechanistic statements as associations.

The alteration in gut microbial community structure and composition induced by parasitic infections is likely due to parasite-associated changes in the gut microenvironment, such as altered intestinal permeability, the availability of nutrients, and gut luminal acidity (pH values). While *Ascaris* species can cause transient tissue damage during larval migration through various organs, their primary role on host pathophysiology is to compromise host nutrition, reducing feed conversion and weight gain in pigs and causing malnutrition and impaired growth in children [33]. A strong link between helminth infection and vitamin A deficiency in preschool-aged children was documented [34]. Depending on burden and developmental stages of worms, the percentage of collective *Ascaris* biomass to the host’s bodyweight is non-trivial. As a result, a direct competition between worms and the host for nutrients, such as glucose and key amino acids, has a negative impact on host physiology [35], which has been documented in pigs [36] and several mouse-parasite systems [37]. Reduced protein utilization and impaired nutrient absorption and transport are modulated by *Ascaris* excretory and secretory (ES) proteins [38]. The electrogenic transport of glucose and alanine by intestinal mucosa was significantly decreased after exposure to total ES proteins. Tissue conductance as an indicator for intestinal permeability was also significantly higher after the addition of adult *A. suum* ES proteins compared to non‑ES controls [38]. Moreover, parasitic infections also disrupt bile acid homeostasis and bile acid signaling in the small intestine, resulting in a significant decrease in both primary, such as α- muricholic acid and chenodeoxycholic acid, and secondary bile acids, such as taurodeoxycholic acids and taurolithocholic acid, in rodent models [39]. The alterations in gut luminal bile acids will further exert the influence of parasites on host mucosal physiology and immunity. Understandably, these mechanistic interpretations are based on associations observed *in vivo* and do not establish causality within the present study.

*Lactobacillus* as the largest genus of lactic acid bacteria has been extensively studied for their application in food preservation, dietary supplements, and biotechnology. The species from this genus can be native inhabitants of the gut microbiota of vertebrates or transiently acquired from the environment and diet. Approximately 34% of healthy humans harbor one or more *Lactobacillus* species in the gut [40]. Moreover, their prevalences display distinct age-specific patterns influenced by geography and are associated with various physiological (such as aging) and pathological conditions, such as colon cancer [40,41]. *Lactobacillus* can metabolize a wide array of substrates, including peptides and acids, and are frequently found in nutrient-rich habitats. *Lactobacillus* species utilize carbohydrates as a primary energy source to produce lactic acid, affecting gut microenvironments in certain gut habitats by lowering luminal pH values. As commensals in human gut microbiota, *Lactobacillus* can enhance intestinal barrier functions [42] and restrict gut colonization of pathogenic bacteria, such as multidrug-resistant *Enterobacteriaceae*, by interacting with *Clostridales*, generating a hostile environment for these pathogens, with reduced key nutrients, such as amino acids and glucose [43]. The abundance of *Lactobacillus* is highly responsive to changes in diets, host factors, and gut microenvironments. For example, dietary carbohydrates are known to increase *Lactobacillus* species in the gut. Moreover, *Lactobacillus* species can adopt various new catabolic pathways to cope with stress, depending on species or functional diversity within this genus. In this study, we were able to detect 19 *Lactobacillus* species using full-length 16S rRNA gene sequencing. While wide inter-individual variations are noticed, *L. reuteri*, *L. johnsonii*, and *L. amylovorus* appear to be among the most abundant, in this order, supporting their ecological role in the gut microbiota as autochthonous species. Collectively at the genus level, *A. suum* infection had little effect on *Lactobacillus*, compared to uninfected and untreated controls. However, FB treatment increased its abundance by 1.74 marginally (*p* = 0.058). However, multiple species or strains, such as *L. reuteri* I5007, were significantly increased by the FB treatment.

As a widely used anthelmintic in the benzimidazole drug class, FB is efficacious in eliminating *A. suum* adult worms in our study. FB is a versatile molecule, possessing antiviral activities [20]. Another member of the class, mebendazole, is known to inhibit the growth of fungi, such as *Cryptococcus neoformans* and *C. gattii*, and to affect the formation of biofilm by the former [44]. The anthelmintics in this class have been investigated as repurposed antimicrobial agents against infections by both Gram-positive and Gram-negative bacteria [45]. In aquatic systems, FB inhibits bacterial metabolism [46]. In parasite-free BPH/5 mice, FB alters gut microbiota in a sex-dependent manner, resulting in significant increase in the abundance of *Verrucomicrobia* with a concomitant reduction in *Actinobacteria*, compared to pre-FB treatment [47]. However, a minimal impact of FB on the gut microbiota has been noted in dogs [19,48]. In the present study, we observed a substantial alteration in infected pigs 13 days after a successful FB treatment, including a significant increase in the abundance of two major classes, *Actinobacteria* and *Bacilli*. Early studies using ^14^C‑labeled FB suggest that up to 50% of originally administrated FB still remained in pig feces 3 days after treatment (https://www.fao.org). The FB elimination in pig feces after 21 days post-administration was only 30% of the initial dose. It is plausible that FB was still present in the porcine gut at necropsy in our study, given reported fecal persistence, although we did not directly quantify FB concentrations. Further, while the biotransformation of FB by host enzymes is well known [49], microbe-mediated biotransformation is poorly understood. In pig manure, FB can be slowly transformed to fenbendazole-sulfoxide, suggesting that microbes may play a role in determining its fate in the environment [50]. Further, microbes in ruminants improve the bioavailability and efficacy of albendazole prodrugs [51]. As a result, it is of importance to have a deep understanding of the interactions between *A. suum* and the anthelmintic drugs under the context of pathological and physiological relevance.

We detected a significant increase in fecal histamine levels after a successful FB treatment in infected pigs. As a versatile molecule with numerous biological properties, including modulating inflammation and regulating gut physiology, histamine can be synthesized in host mast cells and by gut microbes. Diets are also an important source of histamine. Recent studies showed that therapeutics targeting bacterial histamine production can help treat visceral hyperalgesia in certain patients with irritable bowel syndrome [52]. Our qRT-PCR data did not show significant changes in the mRNA expression of mast cell-specific genes, including mast cell-specific carboxypeptidase A3 (*CPA3*) and tryptase beta 2 (*TPSB2*) as well as *HDC*, a gene encoding histidine decarboxylase responsible for the decarboxylation of histidine to form histamine, in the ileum tissue of infected and FB‑treated pigs. Together, these data suggest that the observed increase in histamine in gut luminal contents may result from the gut microbiota. Indeed, our *in vitro* pure culture confirmed this hypothesis. FB in the presence of *A. suum* crude proteins increased histamine production by *L. reuteri* by ~79% after 48 h incubation (*p* < 0.05), consistent with a microbiome contribution. These *in vitro* results are intended as supportive validation and do not replicate the full complexity of the intestinal environment. FB appeared to increase the abundance of *Lactobacillus*, particularly *L. reuteri* strain I5007 and *L. johnsonii* ATCC33200. The former is known to stimulate the expression of β-defensins in the intestinal tissue and improve the gut health of neonatal piglets by increasing colonic butyrate concentration [53]. In addition to *L. reuteri*, at least 17 histamine producing species were detected in the porcine gut microbiota in our study, based on the presence of the *hdc* operon in their genomes [54] and a literature search for experimentally validated histamine production potential of the microbiota.

Prior studies demonstrated that histamine increases intestinal muscle cell contraction in a concentration-dependent manner *in vitro* [55]. Histamine also causes a significant smooth muscle contraction at ≥50 µM in an *ex vivo* mouse model, likely through its receptors H1R and H2R [56]. It is well known that enhanced smooth muscle contraction and intestinal mobility are key contributing factors in self-expulsion of parasites from the host [57^],^ [58]. Moreover, histamine signaling through H1R and H2R increases the susceptibility of mice to infection with the parasite *Plasmodium* and contributes to malaria pathogenesis [59] and affects bacterial infection via altered innate immune responses [60]. Nevertheless, the physiological relevance of microbially derived histamine in this context requires further *in vivo* validation, and causal pathways remain to be established.

Based on a wealth of evidence generated from the present study and those previously published, we formulated the following hypotheses. *A. suum* infection in pigs reduces glucose absorption and impairs the transport of essential amino acids and nucleotides, such as alanine and adenine, altering the gut microenvironment toward nutrient deficiency. The limited nutrient availability represses the expansion of some key species that rely on these nutrients, such as *L. reuteri*. In addition, parasitic infections decrease intestinal lumen contents of multiple primary and secondary bile acids as documented in a rodent model [39], which is also supported by our untargeted metabolome dataset in which primary bile acid biosynthesis and lysis degradation pathways are significantly disrupted by infection. FB treatments successfully eliminate *A. suum* worms and alleviate nutrient stress, allowing certain bacteria, including *Lactobacillus* species, to expand their populations. Various *Lactobacillus* species possess bile salt hydrolase activities and are able to deconjugate bile acids [61]. Many gut microbes can utilize liberated glycine and taurine from conjugated bile acids as nutrient sources. Furthermore, *A. suum* crude proteins are rich in peptides and amino acids, including histidine, the substrate for histamine biosynthesis. At least 17 bacterial species detected in our study possess properties to produce histamine. Histamine produced in turn exerts its biological functions via its regulatory roles in inflammation and smooth muscle contraction, reshaping host–parasite interactions. These hypotheses are proposed to guide future work and are not asserted as causal conclusions in the present cross-sectional study. Our future studies will focus on the pathophysiological implications of the long-lasting changes in gut microbial communities and their metabolic products and the understanding of key microbiota factors in modifying host–parasite interactions.

In conclusion, Ascaris roundworms are among the most important parasites to human health and swine production. In this study, we examined the potential physiological relevance of antiparasitic treatments by a popular dewormer, fenbendazole, using a pig model. Ascaris infection had a substantial impact on microbial composition and serum and fecal metabolites. The fenbendazole treatment eliminated all worms from the gut. Nevertheless, over a dozen of bacterial species still differed substantially in relative abundance between the time-matched normal infected pigs and those successfully treated pigs, 13 days after worm clearance. Eleven pathways were also enriched in successfully treated pigs, including peptidoglycan biosynthesis and histidine metabolism, suggesting that infection-induced alterations can be long-lasting. The interactions between parasite proteins and fenbendazole strongly stimulated histamine production by *L. reuteri in vitro*. Our data support associations between microbiota dynamics, metabolite profiles, and host–parasite interactions *in vivo*, with *in vitro* findings serving as complementary validation. Interpretations of treatment effects are made cautiously due to the absence of an uninfected drug-only group and longitudinal sampling. Our understanding on complex relations between the gut microbiota, the host, and parasites will be conducive to the design of next-generation functional anthelmintics.

## Materials and methods

### Animal care and parasitology

Thirty weaned female pigs (breed: White Yorkshire × Landrace), aged between 6 and 7 months, were obtained from Oak Hill Genetics (Ewing, IL, USA). Upon arrival, the pigs were maintained indoors on sealed concrete floors throughout the experiment and fed ad lib a balanced corn/soybean meal ration containing 16% protein and vitamins and minerals that exceeded National Research Council guidelines and with free access to water. Animals were handled exactly in accordance with the procedures approved by the Beltsville Area Animal Care and Use Committee (Animal Use Protocol #20–003). The Institutional Animal Care and Use Committee (IACUC) guidelines, mandated by federal laws such as the Animal Welfare Act and the Health Research Extension Act, were strictly adhered to. After 1 week’s acclimation, 20 of the pigs were inoculated with a single dose of ~10,000 *A. suum* infective eggs (Batch# 19–01) via oral gavage while 10 pigs served as uninfected controls. At 21 dpi, one group of the infected pigs received a single daily oral dose of Panacur (fenbendazole at 5 mg/kg body weight/day) for three consecutive days (IP) while another group of infected pigs received PBS as untreated controls (IU). The uninfected pigs remained untreated throughout the experiment (NU). Given constraints on animal facilities and resources, our design did not include an uninfected FB-only group or longitudinal sampling, and we therefore interpreted drug-associated changes with caution. Thirteen days after the last of the three Panacur doses (34 dpi), all pigs were sacrificed. The euthanasia procedures were carried out in accordance with the most recent American Veterinary Medical Association Guidelines on Euthanasia for swine. No other animals were present in the same room where euthanasia took place. Briefly, the euthanasia solution (Euthasol®) was administered by i.v. injection at recommended dose for the weight of the pig. For pigs with bodyweight <40 kg, one person placed the pig supine in a V-shaped trough and manually held down the lower jaw and rear legs at the hip or a restraining snare will be placed in the mouth and around the nose in an upright position for pigs with bodyweight > 40 kg; the second person will insert a 16 gauge 1.5 – 2.5 inch needle (depending on the age of the pig) into the right jugular fossa (vein) that was skin surface-swabbed with an alcohol saturated pad to aseptically inject the amount of Euthasol prescribed by the manufacturer. Pigs administered with a correct amount of euthanasia solution were exsanguinated by severing the great vessels in the axillary and inguinal region to prevent blood contamination in the lungs that would result if the neck were severed as the secondary method of euthanasia. Death is confirmed by lack of vital signs (heart rate, respiratory rate, and lack of corneal reflex) prior to proceeding to the secondary method. At necropsy, serum samples were collected for untargeted metabolomics. The pathology of the liver and the intestine was visually inspected and recorded at necropsy. The entire intestine contents were collected for *A. suum* worm counts. The sex, length, and developing stages of all worms were determined. Luminal contents from the proximal colon were also collected at necropsy for microbiome studies and untargeted metabolome analysis. The ileum tissue adjacent to the cecum was dissected and placed in TRIzol reagent and snap-frozen in liquid nitrogen for total RNA extraction. Approximately 1 g of fresh feces was collected and placed in a BIOME-Preserve Microbiome Collection kit (Anaerobe Systems, Morgan Hill, CA, USA) for anaerobic culture.

### Untargeted metabolomic analysis

The untargeted metabolomic analysis of both serum and colon luminal content samples collected at necropsy was conducted separately as previously described [62]. Briefly, serum samples were carefully measured, mixed with methanol at 1:3 (v/v), and vortexed. For fecal samples (colon luminal contents), 800 µl of 80% methanol was added to 50 mg samples, vortexed, ground, and then sonicated. After extraction at –20 °C for 1 h, both serum and fecal mixtures were centrifuged at 12,000 g at 4°C for 15 min. 5 µL of DL-o-chlorophenylalanine at 0.14 mg/mL was added to 200 µL of the supernatant, mixed, and transferred to a new vial for LC-MS analysis using an UPLC – tandem mass spectrometer (UPLC–MS/MS), which consists of an Acquity UPLC System (Waters, Milford, MA, USA) and a Q Exactive high‑resolution/accurate mass spectrometer (Thermo Scientific, Waltham, MA, USA) interfaced with a heated electrospray ionization source (ESI). Both ESI+ and ESI- modes were used to ionize compounds. The raw spectrum data were collected and then aligned using Compound Discoverer software (v3.1) based on the mass/charge ratio (m/z) and the retention time of the ion signals for the initial identification of metabolites. The Human Metabolome Database (HMDB) was used for metabolite annotation based on input sample types [63]. The peak intensity data were normalized based on the median and log‑transformed and then analyzed using a non‑parametric Mann‑Whitney U test to identify metabolites that may differ significantly between experimental groups. The origin of the metabolites identified in serum and fecal samples was derived using the MetOrigin algorithm [22].

### Full-length 16S rRNA gene sequencing-based microbiome study

Colon luminal contents (feces) were collected at necropsy. Total DNA was extracted from fecal samples using a QIAamp Fast DNA Stool Mini kit (Qiagen) with modifications [64]. A bead-beating procedure using a FastPrep 5 G instrument and Lysing Matrix E was performed. Lysis at 70°C was extended to 8 min. Both nuclease‑free water as a non-DNA template control and the microbiome standard D6331 (Zymo Research, Irvine, CA, USA) were processed along with experimental samples following the same protocol and parameters to validate the workflow. The full‑length 16S rRNA gene was amplified and sequenced based on LoopSeq long-read chemistry (Element BioSciences, San Diego, CA, USA) using an Illumina NovoSeq 6000 sequencer. The raw reads were first trimmed using Trimmomatic (v0.39) and then de novo assembled into full-length 16S rRNA gene sequences using the SPAdes algorithm in the LoopSeq proprietary pipeline. The resultant full-length 16S rRNA gene sequences were analyzed using the DADA2 algorithm (v1.16) to generate amplicon sequence variant (ASV) feature tables [65]. The ASV data were then analyzed as previously described [66]. The SILVA ‎Small Subunit (SSU) rRNA Database (release v138) was used for bacterial species/strain annotation. Raw sequence data have been deposited to the NCBI SRA database (SRA accession/BioProject: PRJNA1156568).

### Gene expression analysis using real-time RT-PCR

Total RNA was extracted from ileum tissue adjacent to the cecum using TRIzol reagents (Thermofisher, Carlsbad, CA, USA), treated with DNase I, and then purified using RNA Clean and Concentrator-25 (Zymo Research, Irvine, CA, USA). RNA integrity was verified using a BioAnalyzer 2100 (Agilent, Palo Alto, CA, USA). Quantitative RT-PCR reactions were carried out in a CFX Connect Real-Time PCR Detection System. A standard-curve based absolute quantification method was used [64].

### Bacterial strains, fecal culture conditions, and histamine analysis

*L. reuteri* (strain F275) was purchased from ATCC and cultured in deMan, Rogosa, Sharpe (MRS) media (ATCC medium 416) under aerobic conditions as instructed at 37°C for 24 – 48 h. The fecal inoculum was prepared from homogenized fecal samples preserved in a BIOME preservation kit and then inoculated into LDMIII, a semi-defined medium [52]. Each fecal culture was grown at 37°C for 48 h in a Whitley DG250 anaerobic workstation (Microbiology International, Frederick, MD) supplied with a mixture of 10% CO_2_, 10% H_2_, and 80% N_2_. The cells were pelleted (12000×*g*, 5 min) after 24 h or 48 h incubation. Bacterial cell-free supernatants were used for histamine determination using a histamine ELISA kit (Abcam, Waltham, MA, USA).

### Statistical and bioinformatic analyses

The R Stats package (R version 4.3.0) was used for statistical analyses. A non-parametric Wilcoxon Rank Sum (Mann–Whitney U test) was used for two-group statistical comparisons. Numbers were expressed as means ± SD, unless stated otherwise. The *p* value ≤0.05 was considered statistically significant. For microbiome analysis, the significance threshold was considered at false discovery rate (FDR)-adjusted *q* values ≤0.05. FDR corrections were also applied to unadjusted *p* values for metabolomic analyses, whereby the significance threshold was also FDR-adjusted *q* values ≤0.05. For integrated multi-omics analysis, the DIABLO [23] and MixOmics [67] algorithms were used.

## Supplementary Material

20260209_Ascaris supplementary information.docx

## Data Availability

The datasets generated and/or analyzed during the current study are available in the NCBI SRA repository (www.ncbi.nlm.nih.gov/bioproject/PRJNA1156568). The metabolome dataset can be freely accessed via Mendeley Data (https://data.demo-uni.com/datasets/kz72byft8v/3). DOI for this Mendeley dataset is DOI:10.17632/kz72byft8v.3.
