# Supplementary material for: In vivo porcine multi-omics integration identifies microbiome-driven histamine elevation and lasting gut perturbations following Ascaris suum infection and fenbendazole treatment
Source: Virulence. 2026 Sep 11;17(1):2728766. doi: 10.1080/21505594.2026.2728766 (PMC13577283; doi:10.1080/21505594.2026.2728766)
Supplement: Supplementary file 1 [file KVIR_A_2728766_SM2134.docx]

**Supplementary information**

**The interactions between the roundworm *Ascaris suum* and the antiparasitic drug fenbendazole increased histamine production by *Lactobacillus* in porcine models**

Fang Liu, Hannah E. Streett, Gloria Solano-Aguilar, Allen D. Smith, Donald Carbaugh, Joseph F. Urban Jr., Robert W. Li

**Figure S1.** The effect of *Ascaris suum* infection and subsequent anthelmintic treatment on the gut microbial community in the proximal colon.

A. Alpha diversity (Simpson Index). **B.** Bacteroidaceae. *C.* Caloramatoraceae. **D.** Propionibacteriaceae. **E.** Spirochaetaceae. **F.** Streptococcaceae.

NU: Normal uninfected and untreated pigs; IU: Infected and untreated pigs. IP: Infected and fenbendazole treated pigs.

**P* < 0.05; ** *P* < 0.01; *** *P* < 0.001; *N* = 10 per group.

**Figure S2.** The Random Forest classification model identified the most important serum metabolites distinguishing the status of the infection (A) and the anthelmintic treatment (B). NU: Uninfected and untreated controls. IU: Infected and untreated pigs. IP: Infected and fenbendazole treated pigs. The number and color in the square denote correlation coefficients (r). *N* = 10 per group**.**

**Figure S3.** The short-chain fatty acid (SCFA) concentration in proximal colon contents in pigs in response to Ascaris suum infection and fenbendazole treatment. NU: Uninfected and untreated controls. IU: Infected and untreated pigs. IP: Infected and fenbendazole treated pigs. *N* = 10 per group**.** * *P* < 0.05.

**Figure S4.** The spearman correlation between select fecal metabolites and fecal microbiome features. The vertical bars on the left represent the origin of the fecal metabolites predicted using the MetOrigin algorithm (v2.0). NU: Uninfected and untreated controls. IU: Infected and untreated pigs. IP: Infected and fenbendazole treated pigs. The number and color in the square denote correlation coefficients (r). *N* = 10 per group.

**Figure S5.** An arrow plot generated using multiblock sparse partial least squares discriminant analysis (sPLS-DA) model. The samples are projected into the space spanned by the first two components for each data block, highlighting the agreement between datasets at the sample level. Arrows further away from their centroids indicates some disagreement between the data blocks. NU: Uninfected and untreated controls. IU: Infected and untreated pigs. IP: Infected and fenbendazole treated pigs.

**Figure S6.** A circos plot depicting the strong correlation of select variables between multi-omics data blocks. The variables were selected using DIALBO for serum metabolites (Smetabolism), fecal metabolites (Fmetabolism) and fecal microbiome taxa (Microbiota). The Red: positive correlation; The blue line: negative correlation. The curves outside the ideogram represents the average value of a given variable in a data block. Only the correlation ( r ) ≥ 0.7 are shown. NU: Uninfected and untreated controls. IU: Infected and untreated pigs. IP: Infected and fenbendazole treated pigs. *N* = 10 per group.

**Table S1.** Serum metabolites significantly dysregulated during *Ascaris suum* infection and subsequent the anthelmintic drug treatment. NU: Uninfected and untreated controls. The number denotes normalized counts. IU: Infected and untreated pigs. IP: Infected and fenbendazole treated pigs. *N* = 10 per group. *P* values were based on the Mann–Whitney U test. ESI: Electrospray Ionization.

| **Metabolite**  **ID** | **Metabolite**  **name** | **Metabolite Origin** | **P value** | **Detection mode** | **NU mean** | **NU**  **sd** | **IU**  **mean** | **IU**  **sd** | **IP**  **mean** | **IP**  **sd** | **Contrast** |
| --- | --- | --- | --- | --- | --- | --- | --- | --- | --- | --- | --- |
| HMDB0001257 | Spermidine | Cometabolism | 0.0005 | Positive | 41.97 | 24.14 | 125.42 | 66.98 | 35.45 | 28.62 | NU vs IU |
| HMDB0000181 | L-Dopa | Cometabolism | 0.0007 | Positive | 11.28 | 2.27 | 16.36 | 3.20 | 16.84 | 2.83 | NU vs IU |
| HMDB0000161 | Alanine | Cometabolism | 0.0232 | Positive | 758.18 | 338.41 | 1044.64 | 216.20 | 785.06 | 241.36 | NU vs IU |
| HMDB0000277 | Sphingosine 1-phosphate | Cometabolism | 0.0288 | Positive | 335.85 | 110.35 | 449.74 | 123.67 | 315.91 | 110.41 | NU vs IU |
| HMDB0000177 | L-Histidine | Cometabolism | 0.0185 | Positive | 516.50 | 128.25 | 695.35 | 151.45 | 532.83 | 140.24 | NU vs IU |
| HMDB0000252 | Sphingosine | Cometabolism | 0.0433 | Positive | 84.22 | 31.75 | 117.28 | 25.57 | 165.23 | 261.52 | NU vs IU |
| HMDB0061154 | Monic acid | Drug related | 0.0068 | Positive | 40.21 | 23.20 | 80.82 | 35.70 | 43.70 | 17.25 | NU vs IU |
| HMDB0060549 | Norketamine | Drug related | 0.0433 | Positive | 143.90 | 106.86 | 249.69 | 124.05 | 252.57 | 129.88 | NU vs IU |
| HMDB0006203 | 5-alpha-Dihydrotestosterone glucuronide | Drug related | 0.0052 | Positive | 54.54 | 23.86 | 90.47 | 25.97 | 69.05 | 43.45 | NU vs IU |
| HMDB0000784 | Azelaic acid | Drug related | 0.0185 | Positive | 932.30 | 730.89 | 1322.19 | 489.31 | 934.47 | 435.19 | NU vs IU |
| HMDB0014917 | Nalidixic Acid | Drug related | 0.0433 | Positive | 107.18 | 66.08 | 161.94 | 96.45 | 114.21 | 51.46 | NU vs IU |
| HMDB0000670 | Homoarginine | Drug related | 0.0355 | Positive | 52.45 | 12.59 | 68.83 | 18.11 | 46.93 | 4.80 | NU vs IU |
| HMDB0000824 | Propionylcarnitine | Food related | 0.0021 | Positive | 61.59 | 22.87 | 182.74 | 92.19 | 68.49 | 28.45 | NU vs IU |
| HMDB0010378 | 5,8,11-Eicosatrienoic acid | Food related | 0.0089 | Positive | 20.78 | 8.53 | 32.42 | 8.77 | 23.98 | 9.60 | NU vs IU |
| HMDB0037831 | 8-Hydroxyhexadecanedioic acid | Food related | 0.0089 | Positive | 167.15 | 50.76 | 221.75 | 40.45 | 163.51 | 45.59 | NU vs IU |
| HMDB0002040 | 4-Methoxycinnamic acid | Food related | 0.0433 | Positive | 3.40 | 0.78 | 4.13 | 1.16 | 3.19 | 0.67 | NU vs IU |
| HMDB0000821 | Phenylacetylglycine | Host | 0.0433 | Positive | 1776.23 | 805.26 | 2560.56 | 409.87 | 2928.42 | 705.68 | NU vs IU |
| HMDB0000857 | Pimelic acid | Microbiota | 0.0089 | Positive | 57.51 | 110.45 | 92.61 | 59.86 | 62.30 | 56.05 | NU vs IU |
| HMDB0000883 | Valine | Cometabolism | 0.0232 | Positive | 11117.47 | 2687.12 | 8659.66 | 2243.75 | 9676.65 | 1518.95 | NU vs IU |
| HMDB0000086 | Glycerophosphocholine | Cometabolism | 0.0029 | Positive | 782.77 | 210.49 | 477.47 | 203.46 | 572.64 | 325.51 | NU vs IU |
| HMDB0000510 | Aminoadipic acid | Cometabolism | 0.0433 | Positive | 410.26 | 281.72 | 209.27 | 104.23 | 287.46 | 161.60 | NU vs IU |
| HMDB0000062 | Carnitine | Drug related | 0.0355 | Positive | 2244.78 | 757.79 | 1650.84 | 638.24 | 1838.79 | 409.43 | NU vs IU |
| HMDB0003229 | Palmitoleic acid | Drug related | 0.0029 | Positive | 97.35 | 81.78 | 25.76 | 12.63 | 39.41 | 19.42 | NU vs IU |
| HMDB0002000 | Myristoleic acid | Food related | 0.0232 | Positive | 52.80 | 21.83 | 38.20 | 4.42 | 34.18 | 5.84 | NU vs IU |
| HMDB0011172 | gamma-Glutamylvaline | Food related | 0.0029 | Positive | 199.15 | 109.64 | 121.17 | 22.59 | 176.25 | 30.30 | NU vs IU |
| HMDB0011517 | LysoPE(20:4(5Z,8Z,11Z,14Z)/0:0) | Food related | 0.0232 | Positive | 195.31 | 75.39 | 129.19 | 45.15 | 175.74 | 80.71 | NU vs IU |
| HMDB0004705 | 12,13-DHOME | Food related | 0.0089 | Positive | 166.92 | 85.63 | 85.28 | 28.85 | 95.21 | 32.29 | NU vs IU |
| HMDB0062549 | 2-Hydroxystearic acid | Food related | 0.0355 | Positive | 173.30 | 159.10 | 64.25 | 17.48 | 70.45 | 34.87 | NU vs IU |
| HMDB0006270 | Linoelaidic acid | Food related | 0.0433 | Positive | 136.39 | 138.34 | 51.65 | 38.98 | 75.12 | 47.45 | NU vs IU |
| HMDB0060038 | 10Z-Heptadecenoic acid | Food related | 0.0185 | Positive | 6.30 | 3.56 | 2.88 | 1.88 | 6.38 | 4.06 | NU vs IU |
| HMDB0010733 | 3-Oxohexadecanoic acid | Food related | 0.0089 | Positive | 34.10 | 31.59 | 8.60 | 3.38 | 12.83 | 7.75 | NU vs IU |
| HMDB0004702 | 12,13-EpOME | Host | 0.0232 | Positive | 170.39 | 104.51 | 81.22 | 34.77 | 106.62 | 72.81 | NU vs IU |
| HMDB0013279 | N-Nonanoylglycine | Unknown | 0.0232 | Positive | 106.86 | 45.52 | 74.42 | 35.55 | 83.09 | 35.99 | NU vs IU |
| HMDB0000127 | Glucuronic acid | Cometabolism | 0.0185 | Negative | 26.29 | 24.80 | 51.64 | 29.25 | 53.05 | 25.67 | NU vs IU |
| HMDB0001383 | Sphinganine 1-phosphate | Cometabolism | 0.0288 | Negative | 790.34 | 551.46 | 1241.95 | 618.83 | 618.01 | 357.43 | NU vs IU |
| HMDB0000044 | Ascorbic acid | Cometabolism | 0.0185 | Negative | 18.53 | 11.51 | 32.52 | 12.38 | 31.92 | 8.14 | NU vs IU |
| HMDB0000197 | Indoleacetic acid | Cometabolism | 0.0355 | Negative | 36.93 | 10.98 | 73.86 | 62.28 | 35.92 | 15.93 | NU vs IU |
| HMDB0000159 | L-Phenylalanine | Cometabolism | 0.0433 | Negative | 5239.71 | 1091.92 | 6206.36 | 1108.54 | 5098.19 | 1247.17 | NU vs IU |
| HMDB0010410 | Resolvin E1 | Drug related | 0.0068 | Negative | 21.95 | 8.15 | 45.37 | 19.60 | 36.23 | 11.63 | NU vs IU |
| HMDB0029885 | Sorbitan laurate | Drug related | 0.0029 | Negative | 20.92 | 7.39 | 41.16 | 20.07 | 23.96 | 6.08 | NU vs IU |
| HMDB0014554 | Mupirocin | Drug related | 0.0007 | Negative | 173.18 | 54.30 | 281.06 | 59.28 | 208.09 | 91.60 | NU vs IU |
| HMDB0000784 | Azelaic acid | Drug related | 0.0068 | Negative | 4236.85 | 3678.78 | 5907.02 | 2084.35 | 4064.91 | 1945.16 | NU vs IU |
| HMDB0006203 | 5-alpha-Dihydrotestosterone glucuronide | Drug related | 0.0003 | Negative | 46.98 | 7.61 | 69.31 | 17.48 | 49.59 | 19.76 | NU vs IU |
| HMDB0000666 | Heptanoic acid | Drug related | 0.0005 | Negative | 130.78 | 32.50 | 191.52 | 29.28 | 165.16 | 51.46 | NU vs IU |
| HMDB0000355 | 3-Hydroxymethylglutaric acid | Drug related | 0.0021 | Negative | 903.51 | 132.45 | 1149.45 | 193.84 | 948.98 | 113.21 | NU vs IU |
| HMDB0000792 | Sebacic acid | Drug related | 0.0052 | Negative | 1205.70 | 112.10 | 1449.35 | 207.05 | 1271.41 | 154.13 | NU vs IU |
| HMDB0003252 | Thromboxane B2 | Food related | 0.0015 | Negative | 16.28 | 8.64 | 40.58 | 21.46 | 17.57 | 8.53 | NU vs IU |
| HMDB0013623 | 12(13)Ep-9-KODE | Food related | 0.0011 | Negative | 125.20 | 41.00 | 209.10 | 49.21 | 136.92 | 48.37 | NU vs IU |
| HMDB0037831 | 8-Hydroxyhexadecanedioic acid | Food related | 0.0089 | Negative | 300.09 | 72.34 | 387.27 | 78.58 | 279.40 | 52.08 | NU vs IU |
| HMDB0031740 | Methyl dihydrojasmonate | Food related | 0.0288 | Negative | 63.48 | 13.44 | 88.95 | 27.03 | 73.35 | 9.47 | NU vs IU |
| HMDB0000888 | Undecanedioic acid | Food related | 0.0232 | Negative | 387.05 | 61.35 | 479.16 | 85.87 | 394.84 | 32.41 | NU vs IU |
| HMDB0011635 | p-Cresol sulfate | Microbiota | 0.0001 | Negative | 1434.63 | 620.54 | 4209.63 | 2302.17 | 2325.88 | 873.00 | NU vs IU |
| HMDB0000857 | Pimelic acid | Microbiota | 0.0185 | Negative | 303.14 | 464.37 | 393.92 | 207.94 | 301.34 | 214.95 | NU vs IU |
| HMDB0001873 | Isobutyric acid | Microbiota | 0.0115 | Negative | 7.89 | 2.53 | 23.26 | 26.65 | 8.46 | 2.89 | NU vs IU |
| HMDB0000350 | 3-Hydroxysebacic acid | Unknown | 0.0433 | Negative | 534.87 | 245.21 | 740.16 | 256.08 | 498.50 | 109.98 | NU vs IU |
| HMDB0001448 | Sulfate | Cometabolism | 0.0089 | Negative | 3529.96 | 866.04 | 2380.47 | 837.76 | 3223.71 | 1063.89 | NU vs IU |
| HMDB0000207 | Oleic acid | Cometabolism | 0.0185 | Negative | 1225.31 | 761.15 | 452.53 | 200.67 | 833.45 | 659.95 | NU vs IU |
| HMDB0001388 | alpha-Linolenic acid | Cometabolism | 0.0433 | Negative | 745.46 | 671.45 | 231.45 | 165.95 | 431.32 | 439.93 | NU vs IU |
| HMDB0000806 | Myristic acid | Cometabolism | 0.0068 | Negative | 219.56 | 165.10 | 59.37 | 21.16 | 104.59 | 55.21 | NU vs IU |
| HMDB0003229 | Palmitoleic acid | Drug related | 0.0433 | Negative | 651.31 | 547.10 | 200.36 | 124.45 | 339.01 | 254.74 | NU vs IU |
| HMDB0034297 | Ricinoleic acid | Drug related | 0.0068 | Negative | 1310.49 | 937.74 | 415.89 | 222.94 | 637.66 | 303.66 | NU vs IU |
| HMDB0011496 | LysoPE(0:0/22:6(4Z,7Z,10Z,13Z,16Z,19Z)) | Food related | 0.0355 | Negative | 63.83 | 33.67 | 40.10 | 14.04 | 55.80 | 32.82 | NU vs IU |
| HMDB0059633 | (9S,10S)-9,10-dihydroxyoctadecanoate | Food related | 0.0355 | Negative | 293.82 | 132.04 | 191.23 | 54.27 | 192.54 | 76.72 | NU vs IU |
| HMDB0006528 | Docosapentaenoic acid (22n-3) | Food related | 0.0288 | Negative | 32.74 | 13.68 | 18.92 | 7.38 | 31.81 | 14.06 | NU vs IU |
| HMDB0011517 | LysoPE(20:4(5Z,8Z,11Z,14Z)/0:0) | Food related | 0.0355 | Negative | 1061.87 | 490.53 | 711.94 | 260.74 | 987.31 | 461.21 | NU vs IU |
| HMDB0002226 | Adrenic acid | Food related | 0.0355 | Negative | 65.53 | 47.21 | 30.22 | 12.24 | 41.49 | 25.74 | NU vs IU |
| HMDB0061690 | LysoPI(20:4(5Z,8Z,11Z,14Z)/0:0) | Food related | 0.0115 | Negative | 439.98 | 169.10 | 255.51 | 86.87 | 383.11 | 208.26 | NU vs IU |
| HMDB0061695 | LysoPI(16:0/0:0) | Food related | 0.0068 | Negative | 63.89 | 27.48 | 36.02 | 21.12 | 66.97 | 35.92 | NU vs IU |
| HMDB0004705 | 12,13-DHOME | Food related | 0.0185 | Negative | 451.86 | 238.65 | 241.95 | 87.89 | 254.87 | 108.05 | NU vs IU |
| HMDB0006270 | Linoelaidic acid | Food related | 0.0355 | Negative | 2013.03 | 1366.37 | 858.03 | 375.73 | 1170.82 | 667.58 | NU vs IU |
| HMDB0005821 | Beta-Cortol | Food related | 0.0288 | Negative | 46.59 | 33.77 | 21.42 | 7.02 | 26.08 | 10.52 | NU vs IU |
| HMDB0002259 | Heptadecanoic acid | Food related | 0.0115 | Negative | 65.43 | 37.47 | 29.43 | 4.59 | 42.39 | 33.67 | NU vs IU |
| HMDB0005060 | Eicosadienoic acid | Food related | 0.0288 | Negative | 74.42 | 50.20 | 26.32 | 13.49 | 44.57 | 31.57 | NU vs IU |
| HMDB0034153 | Ethyl tetradecanoate | Food related | 0.0355 | Negative | 469.89 | 321.35 | 182.99 | 57.00 | 246.67 | 113.07 | NU vs IU |
| HMDB0062549 | 2-Hydroxystearic acid | Food related | 0.0068 | Negative | 1149.27 | 902.62 | 408.23 | 152.01 | 494.00 | 235.73 | NU vs IU |
| HMDB0061657 | 3-hydroxypentadecanoic acid | Food related | 0.0039 | Negative | 20.74 | 16.50 | 6.60 | 3.01 | 9.73 | 7.08 | NU vs IU |
| HMDB0060038 | 10Z-Heptadecenoic acid | Food related | 0.0068 | Negative | 73.15 | 49.17 | 24.80 | 8.45 | 41.49 | 31.62 | NU vs IU |
| HMDB0002261 | 2-Hydroxymyristic acid | Food related | 0.0089 | Negative | 94.00 | 73.24 | 32.56 | 15.56 | 41.93 | 25.22 | NU vs IU |
| HMDB0062436 | 9Z-Eicosenoic acid | Food related | 0.0115 | Negative | 599.84 | 498.91 | 185.31 | 156.68 | 348.08 | 298.10 | NU vs IU |
| HMDB0010733 | 3-Oxohexadecanoic acid | Food related | 0.0021 | Negative | 119.87 | 88.75 | 30.60 | 25.34 | 52.65 | 30.66 | NU vs IU |
| HMDB0006294 | 16-Hydroxy hexadecanoic acid | Food related | 0.0015 | Negative | 760.50 | 634.41 | 161.95 | 66.54 | 266.32 | 123.85 | NU vs IU |
| HMDB0006547 | Stearidonic acid | Host | 0.0288 | Negative | 48.78 | 56.80 | 5.30 | 6.33 | 25.55 | 51.60 | NU vs IU |
| HMDB0059921 | Trimetaphosphoric acid | Unknown | 0.0433 | Negative | 52.74 | 33.83 | 26.25 | 7.93 | 38.30 | 10.08 | NU vs IU |
| HMDB0000532 | Acetylglycine | Unknown | 0.0232 | Negative | 40.64 | 38.39 | 14.53 | 4.57 | 17.11 | 9.68 | NU vs IU |
| HMDB0013622 | 10Z-Nonadecenoic acid | Unknown | 0.0115 | Negative | 36.44 | 22.45 | 12.98 | 4.65 | 24.86 | 26.14 | NU vs IU |
| HMDB0013622 | 10Z-Nonadecenoic acid | Unknown | 0.0433 | Positive | 5.57 | 2.24 | 4.13 | 3.20 | 6.87 | 4.54 | IU vs IP |
| HMDB0000678 | Isovalerylglycine | Unknown | 0.0232 | Positive | 85.74 | 38.32 | 113.29 | 42.04 | 182.89 | 72.96 | IU vs IP |
| HMDB0060038 | 10Z-Heptadecenoic acid | Food related | 0.0147 | Positive | 6.30 | 3.56 | 2.88 | 1.88 | 6.38 | 4.06 | IU vs IP |
| HMDB0031404 | Cyclohexylamine | Food related | 0.0433 | Positive | 1198.66 | 175.25 | 1191.19 | 333.04 | 1551.79 | 422.77 | IU vs IP |
| HMDB0011172 | gamma-Glutamylvaline | Food related | 0.0001 | Positive | 199.15 | 109.64 | 121.17 | 22.59 | 176.25 | 30.30 | IU vs IP |
| HMDB0000070 | Pipecolic acid | Cometabolism | 0.0433 | Positive | 1174.10 | 169.98 | 1132.77 | 304.09 | 1429.35 | 191.28 | IU vs IP |
| HMDB0000033 | Carnosine | Cometabolism | 0.0433 | Positive | 357.00 | 137.45 | 280.97 | 101.33 | 212.04 | 67.76 | IU vs IP |
| HMDB0000252 | Sphingosine | Cometabolism | 0.0115 | Positive | 84.22 | 31.75 | 117.28 | 25.57 | 165.23 | 261.52 | IU vs IP |
| HMDB0000161 | Alanine | Cometabolism | 0.0232 | Positive | 758.18 | 338.41 | 1044.64 | 216.20 | 785.06 | 241.36 | IU vs IP |
| HMDB0000177 | L-Histidine | Cometabolism | 0.0232 | Positive | 516.50 | 128.25 | 695.35 | 151.45 | 532.83 | 140.24 | IU vs IP |
| HMDB0000277 | Sphingosine 1-phosphate | Cometabolism | 0.0232 | Positive | 335.85 | 110.35 | 449.74 | 123.67 | 315.91 | 110.41 | IU vs IP |
| HMDB0001388 | alpha-Linolenic acid | Cometabolism | 0.0068 | Positive | 118.70 | 55.64 | 102.28 | 20.51 | 71.56 | 18.91 | IU vs IP |
| HMDB0001257 | Spermidine | Cometabolism | 0.0011 | Positive | 41.97 | 24.14 | 125.42 | 66.98 | 35.45 | 28.62 | IU vs IP |
| HMDB0000670 | Homoarginine | Drug related | 0.0011 | Positive | 52.45 | 12.59 | 68.83 | 18.11 | 46.93 | 4.80 | IU vs IP |
| HMDB0000784 | Azelaic acid | Drug related | 0.0288 | Positive | 932.30 | 730.89 | 1322.19 | 489.31 | 934.47 | 435.19 | IU vs IP |
| HMDB0006203 | 5-alpha-Dihydrotestosterone glucuronide | Drug related | 0.0433 | Positive | 54.54 | 23.86 | 90.47 | 25.97 | 69.05 | 43.45 | IU vs IP |
| HMDB0061154 | Monic acid | Drug related | 0.0052 | Positive | 40.21 | 23.20 | 80.82 | 35.70 | 43.70 | 17.25 | IU vs IP |
| HMDB0011152 | LysoPE(P-16:0/0:0) | Food related | 0.0089 | Positive | 32.97 | 5.16 | 37.47 | 3.22 | 31.49 | 5.25 | IU vs IP |
| HMDB0000933 | Traumatic acid | Food related | 0.0089 | Positive | 1815.07 | 270.73 | 1876.32 | 316.95 | 1567.84 | 176.13 | IU vs IP |
| HMDB0000413 | 3-Hydroxydodecanedioic acid | Food related | 0.0089 | Positive | 3334.45 | 675.11 | 3533.22 | 710.84 | 2828.46 | 429.72 | IU vs IP |
| HMDB0000394 | 3-Hydroxytetradecanedioic acid | Food related | 0.0089 | Positive | 4835.48 | 1196.53 | 5426.54 | 747.07 | 4429.37 | 696.45 | IU vs IP |
| HMDB0002040 | 4-Methoxycinnamic acid | Food related | 0.0185 | Positive | 3.40 | 0.78 | 4.13 | 1.16 | 3.19 | 0.67 | IU vs IP |
| HMDB0013623 | 12(13)Ep-9-KODE | Food related | 0.0232 | Positive | 62.68 | 21.61 | 76.14 | 22.31 | 53.76 | 16.07 | IU vs IP |
| HMDB0037831 | 8-Hydroxyhexadecanedioic acid | Food related | 0.0039 | Positive | 167.15 | 50.76 | 221.75 | 40.45 | 163.51 | 45.59 | IU vs IP |
| HMDB0000623 | Dodecanedioic acid | Food related | 0.0185 | Positive | 61.77 | 33.02 | 107.07 | 53.57 | 56.42 | 34.58 | IU vs IP |
| HMDB0000824 | Propionylcarnitine | Food related | 0.0039 | Positive | 61.59 | 22.87 | 182.74 | 92.19 | 68.49 | 28.45 | IU vs IP |
| HMDB0000671 | Indolelactic acid | Microbiota | 0.0052 | Positive | 120.20 | 53.06 | 132.65 | 34.70 | 103.12 | 80.28 | IU vs IP |
| HMDB0000148 | L-Glutamic acid | Cometabolism | 0.0185 | Negative | 845.12 | 460.44 | 645.07 | 252.67 | 1143.63 | 494.80 | IU vs IP |
| HMDB0000883 | Valine | Cometabolism | 0.0147 | Negative | 533.73 | 314.24 | 296.94 | 76.87 | 473.82 | 176.22 | IU vs IP |
| HMDB0000691 | Malonic acid | Cometabolism | 0.0015 | Negative | 16.80 | 9.88 | 12.25 | 2.37 | 23.66 | 16.01 | IU vs IP |
| HMDB0000254 | Succinic acid | Cometabolism | 0.0433 | Negative | 274.91 | 111.20 | 243.91 | 34.02 | 285.92 | 41.67 | IU vs IP |
| HMDB0006294 | 16-Hydroxy hexadecanoic acid | Food related | 0.0433 | Negative | 760.50 | 634.41 | 161.95 | 66.54 | 266.32 | 123.85 | IU vs IP |
| HMDB0006528 | Docosapentaenoic acid (22n-3) | Food related | 0.0185 | Negative | 32.74 | 13.68 | 18.92 | 7.38 | 31.81 | 14.06 | IU vs IP |
| HMDB0011470 | LysoPE(0:0/14:0) | Food related | 0.0052 | Negative | 39097.66 | 4038.41 | 35881.60 | 2459.22 | 41316.18 | 5743.53 | IU vs IP |
| HMDB0000730 | Isobutyrylglycine | Unknown | 0.0355 | Negative | 49.90 | 27.45 | 51.72 | 31.94 | 109.47 | 61.70 | IU vs IP |
| HMDB0000678 | Isovalerylglycine | Unknown | 0.0185 | Negative | 224.53 | 110.05 | 279.10 | 112.79 | 494.79 | 217.58 | IU vs IP |
| HMDB0059921 | Trimetaphosphoric acid | Unknown | 0.0115 | Negative | 52.74 | 33.83 | 26.25 | 7.93 | 38.30 | 10.08 | IU vs IP |
| HMDB0002311 | 8,9-DiHETrE | Cometabolism | 0.0052 | Negative | 29.64 | 7.15 | 35.34 | 7.19 | 25.58 | 5.57 | IU vs IP |
| HMDB0000159 | L-Phenylalanine | Cometabolism | 0.0433 | Negative | 5239.71 | 1091.92 | 6206.36 | 1108.54 | 5098.19 | 1247.17 | IU vs IP |
| HMDB0000033 | Carnosine | Cometabolism | 0.0147 | Negative | 149.86 | 55.42 | 142.00 | 58.52 | 91.91 | 22.82 | IU vs IP |
| HMDB0000277 | Sphingosine 1-phosphate | Cometabolism | 0.0089 | Negative | 1063.72 | 312.01 | 1486.89 | 524.06 | 893.25 | 364.01 | IU vs IP |
| HMDB0000150 | Gluconolactone | Cometabolism | 0.0185 | Negative | 33.04 | 7.99 | 37.40 | 14.19 | 25.26 | 7.20 | IU vs IP |
| HMDB0000197 | Indoleacetic acid | Cometabolism | 0.0288 | Negative | 36.93 | 10.98 | 73.86 | 62.28 | 35.92 | 15.93 | IU vs IP |
| HMDB0001383 | Sphinganine 1-phosphate | Cometabolism | 0.0115 | Negative | 790.34 | 551.46 | 1241.95 | 618.83 | 618.01 | 357.43 | IU vs IP |
| HMDB0000225 | Oxoadipic acid | Cometabolism | 0.0433 | Negative | 71.22 | 22.65 | 95.23 | 46.71 | 54.65 | 17.60 | IU vs IP |
| HMDB0000355 | 3-Hydroxymethylglutaric acid | Drug related | 0.0232 | Negative | 903.51 | 132.45 | 1149.45 | 193.84 | 948.98 | 113.21 | IU vs IP |
| HMDB0000784 | Azelaic acid | Drug related | 0.0089 | Negative | 4236.85 | 3678.78 | 5907.02 | 2084.35 | 4064.91 | 1945.16 | IU vs IP |
| HMDB0029885 | Sorbitan laurate | Drug related | 0.0115 | Negative | 20.92 | 7.39 | 41.16 | 20.07 | 23.96 | 6.08 | IU vs IP |
| HMDB0006203 | 5-alpha-Dihydrotestosterone glucuronide | Drug related | 0.0147 | Negative | 46.98 | 7.61 | 69.31 | 17.48 | 49.59 | 19.76 | IU vs IP |
| HMDB0000394 | 3-Hydroxytetradecanedioic acid | Food related | 0.0029 | Negative | 9501.57 | 2215.31 | 10582.60 | 2071.17 | 8342.86 | 900.99 | IU vs IP |
| HMDB0002497 | Glycochenodeoxycholate-3-sulfate | Food related | 0.0355 | Negative | 0.82 | 0.15 | 14.42 | 42.75 | 0.80 | 0.20 | IU vs IP |
| HMDB0031897 | Phaseolic acid | Food related | 0.0185 | Negative | 351.71 | 33.75 | 397.90 | 111.10 | 301.00 | 51.52 | IU vs IP |
| HMDB0000888 | Undecanedioic acid | Food related | 0.0115 | Negative | 387.05 | 61.35 | 479.16 | 85.87 | 394.84 | 32.41 | IU vs IP |
| HMDB0000413 | 3-Hydroxydodecanedioic acid | Food related | 0.0005 | Negative | 5149.74 | 913.94 | 6067.81 | 1152.05 | 4368.70 | 493.57 | IU vs IP |
| HMDB0037831 | 8-Hydroxyhexadecanedioic acid | Food related | 0.0007 | Negative | 300.09 | 72.34 | 387.27 | 78.58 | 279.40 | 52.08 | IU vs IP |
| HMDB0000847 | Pelargonic acid | Food related | 0.0002 | Negative | 16.73 | 5.31 | 19.70 | 4.43 | 12.69 | 2.27 | IU vs IP |
| HMDB0000872 | Tetradecanedioic acid | Food related | 0.0433 | Negative | 293.89 | 206.90 | 232.36 | 157.68 | 144.93 | 126.99 | IU vs IP |
| HMDB0007856 | LysoPA(18:2(9Z,12Z)/0:0) | Food related | 0.0029 | Negative | 154.68 | 27.28 | 136.60 | 30.73 | 82.83 | 39.13 | IU vs IP |
| HMDB0003320 | Indole-3-carboxylic acid | Food related | 0.0288 | Negative | 40.13 | 16.24 | 46.19 | 9.04 | 41.15 | 24.22 | IU vs IP |
| HMDB0013623 | 12(13)Ep-9-KODE | Food related | 0.0052 | Negative | 125.20 | 41.00 | 209.10 | 49.21 | 136.92 | 48.37 | IU vs IP |
| HMDB0003252 | Thromboxane B2 | Food related | 0.0021 | Negative | 16.28 | 8.64 | 40.58 | 21.46 | 17.57 | 8.53 | IU vs IP |
| HMDB0000307 | 1beta-Hydroxycholic acid | Food related | 0.0433 | Negative | 108.73 | 130.90 | 88.21 | 54.80 | 49.11 | 74.25 | IU vs IP |
| HMDB0004702 | 12,13-EpOME | Host | 0.0089 | Negative | 370.04 | 190.96 | 271.52 | 54.86 | 200.94 | 64.16 | IU vs IP |
| HMDB0004243 | 12(S)-HPETE | Host | 0.0147 | Negative | 13.48 | 5.71 | 17.02 | 10.49 | 8.52 | 3.29 | IU vs IP |
| HMDB0001873 | Isobutyric acid | Microbiota | 0.0232 | Negative | 7.89 | 2.53 | 23.26 | 26.65 | 8.46 | 2.89 | IU vs IP |
| HMDB0000764 | Hydrocinnamic acid | Microbiota | 0.0185 | Negative | 22.84 | 9.41 | 32.94 | 11.67 | 21.42 | 8.75 | IU vs IP |
| HMDB0011635 | p-Cresol sulfate | Microbiota | 0.0147 | Negative | 1434.63 | 620.54 | 4209.63 | 2302.17 | 2325.88 | 873.00 | IU vs IP |
| HMDB0000748 | 3-Phenyllactic acid | Microbiota | 0.0288 | Negative | 276.82 | 205.34 | 387.90 | 223.23 | 227.61 | 118.31 | IU vs IP |
| HMDB0001895 | Salicylic acid | Microbiota | 0.0288 | Negative | 60.86 | 11.84 | 75.30 | 19.45 | 53.01 | 18.16 | IU vs IP |
| HMDB0000671 | Indolelactic acid | Microbiota | 0.0232 | Negative | 553.40 | 277.55 | 582.89 | 171.36 | 411.92 | 223.43 | IU vs IP |
| HMDB0000755 | Hydroxyphenyllactic acid | Microbiota | 0.0089 | Negative | 942.02 | 619.49 | 1006.77 | 305.79 | 664.90 | 318.39 | IU vs IP |
| HMDB0000682 | Indoxyl sulfate | Microbiota | 0.0433 | Negative | 2152.63 | 1615.99 | 4392.88 | 2922.44 | 1851.31 | 1006.70 | IU vs IP |
| HMDB0000350 | 3-Hydroxysebacic acid | Unknown | 0.0147 | Negative | 534.87 | 245.21 | 740.16 | 256.08 | 498.50 | 109.98 | IU vs IP |
| HMDB0028884 | Histidylglutamic acid | Unknown | 0.0232 | Negative | 109.18 | 86.55 | 142.74 | 48.03 | 80.59 | 60.34 | IU vs IP |

**Table S2.** Pathways significantly enriched among fecal metabolites.

| **Pathway ID** | **Pathway**  **enriched** | ***P***  **value** | **Significance**  **in** | **Example (significant)**  **metabolite** | **Detection mode** | **Metabolite origin** |
| --- | --- | --- | --- | --- | --- | --- |
| ko00340 | Histidine metabolism | 4.87e-04 | Drug treatment | Urocanic acid; Histamine | ESI: positive | Co_metabolism |
| ko00473 | D-Alanine metabolism | 3.16e-03 | Drug treatment | Alanylalanine | ESI: negative | Microbiota |
| ko00073 | Cutin, suberine and wax biosynthesis | 6.67e-03 | Drug treatment | Oleic acid | ESI: positive | Co_metabolism |
| ssc00410 | beta-Alanine metabolism | 1.37e-02 | Drug treatment | β-Alanyl-L-lysine | ESI: positive | host |
| ko00550 | Peptidoglycan biosynthesis | 1.58e-02 | Drug treatment | Alanylalanine | ESI: negative | Microbiota |
| ko00750 | Vitamin B6 metabolism | 2.54e-02 | Drug treatment | 4-Pyridoxic acid | ESI: positive | Co_metabolism |
| ssc00120 | Primary bile acid biosynthesis | 2.99e-02 | Drug treatment | 3α,7α-Dihydroxycoprostanic acid | ESI: negative | Host |
| ko00270 | Cysteine and methionine metabolism | 3.51e-02 | Drug treatment | Cysteine-S-sulfate | ESI: negative | Microbiota |
| ko01040 | Biosynthesis of unsaturated fatty acids | 3.96e-02 | Drug treatment | Oleic acid | ESI: positive | Co_metabolism |
| ko00780 | Biotin metabolism | 4.41e-02 | Drug treatment | Biotin | ESI: negative | Co_metabolism |
| ko00410 | beta-Alanine metabolism | 4.78e-02 | Drug treatment | L-Histidine | ESI: negative | Co_metabolism |
| ko00250 | Ala, Asp & Glu metabolism | 2.04e-03 | Infection status | Succinic acid; N-Acetyl-L-aspartic acid | ESI: negative | Co_metabolism |
| ko00330 | Arginine and proline metabolism | 1.10e-02 | Infection status | 4-Hydroxyproline; Ornithine | ESI: negative | Co_metabolism |
| ko00472 | D-Arg & D-Orn metabolism | 2.24e-02 | Infection status | Ornithine | ESI: negative | Co_metabolism |
| ko00623 | Toluene degradation | 2.33e-02 | Infection status | 4-Hydroxybenzaldehyde | ESI: negative | Microbiota |
| ko00310 | Lysine degradation | 2.89e-02 | Infection status | Glutaric acid | ESI: negative | Microbiota |
| ssc00120 | Primary bile acid biosynthesis | 2.99e-02 | Infection status | 3α,7α-Dihydroxycoprostanic acid | ESI: negative | Host |
| ko00750 | Vitamin B6 metabolism | 3.12e-02 | Infection status | 4-Pyridoxic acid | ESI: positive | Co_metabolism |
| ko00626 | Naphthalene degradation | 3.53e-02 | Infection status | Naphthalene | ESI: positive | Co_metabolism |
| ko00627 | Aminobenzoate degradation | 3.89e-02 | Infection status | 4-Hydroxybenzaldehyde | ESI: negative | Microbiota |
| ko00020 | Citrate cycle (TCA cycle) | 4.91e-02 | Infection status | Succinic acid | ESI: negative | Co_metabolism |
